# A quantitative synthesis of the medicinal ethnobotany of the Malinké of Mali and the Asháninka of Peru, with a new theoretical framework

**DOI:** 10.1186/1746-4269-3-36

**Published:** 2007-12-05

**Authors:** Nathaniel Bletter

**Affiliations:** 1 Institute of Systematic Botany, New York Botanical Garden, New York, USA; 2 Biology, Graduate Center of the City University of New York, New York, USA

## Abstract

**Background:**

Although ethnomedically and taxonomically guided searches for new medicinal plants can improve the percentage of plants found containing active compounds when compared to random sampling, ethnobotany has fulfilled little of its promise in the last few decades to deliver a bounty of new, laboratory-proven medicinal plants and compounds. It is quite difficult to test, isolate, and elucidate the structure and mechanism of compounds from the plethora of new medicinal plant uses described each year with limited laboratory time and resources and the high cost of clinical trials of new drug candidates.

**Methods:**

A new quantitative theoretical framework of mathematical formulas called "relational efficacy" is proposed that should narrow down this search for new plant-derived medicines based on the hypothesis that closely related plants used to treat closely related diseases in distantly related cultures have a higher probability of being effective because they are more likely to be independent discoveries of similar plant compounds and disease mechanisms. A prerequisite to this hypothesis, the idea that empirical testing in traditional medicine will lead to choosing similar medicinal plants and therefore the medicinal flora of two distant cultures will prove to be more similar than their general flora, is tested using resampling statistics on cross-cultural field data of the plants used by the Malinké of Mali and the Asháninka of Peru to treat the diseases malaria, African sleeping sickness, Chagas' disease, leishmaniasis, diabetes, eczema, asthma, and uterine fibroids.

**Results:**

In this case, the similarity of the medicinal floras is found to be significantly greater than the similarity of the general floras, but only when the diseases in question are grouped into the categories of parasitic and autoimmune diseases.

**Conclusion:**

If the central theoretical framework of this hypothesis is shown to be true, it will allow the synthesis of medicinal plant information from around the world to pinpoint the species with the highest potential efficacy to take into the laboratory and analyze further, ultimately saving much field and laboratory time and resources.

Spanish abstract

Las búsquedas que utilizan la etnomedicina y la taxonomía para descubrir nuevas plantas medicinales, pueden aumentar la probabilidad de éxito de encontrar compuestos químicos activos en plantas, en comparación con las búsquedas aleatorias. A pesar de lo anterior, en las últimas décadas, la etnobotánica no ha cumplido con las expectativas de proveer numerosas plantas medicinales y químicos nuevos una vez examinados en el laboratorio. Cada año se describen una plétora de plantas medicinales y sus usos, sin embargo las limitaciones de tiempo y recursos en los laboratorios, unidos al alto coste de los ensayos clínicos de las drogas potenciales, hacen muy difícil probar, aislar, y elucidar la estructura y el mecanismo de los compuestos de estas plantas. Se propone un nuevo marco teórico cuantitativo cuyo fin es focalizar la búsqueda de nueva plantas medicinales. Este marco teórico está basado en la hipótesis que las plantas cercanamente relacionadas, usadas para tratar enfermedades cercanamente relacionadas en culturas distantemente relacionadas, tienen una eficacia potencial más alta, debido a que es más probable que estos hallazgos sean descubrimientos independientes de compuestos químicos similares. Parte de esta hipótesis, que las escogencias racionales se hacen para elegir plantas medicinales similares y que la flora medicinal de dos culturas distantes es más similar que su flora general, se probó usando métodos estadísticos de remuestreo con datos de campo de la comunidad Malinké de Malí y de la Asháninka de Perú, y las enfermedades de paludismo, enfermedad africana del sueño, enfermedad de Chagas, leishmania, diabetes, eczema, asma, y fibromas uterinos. Se encontró, en este caso, que la similitud de las floras medicinales es significativamente mayor a la similitud de las floras generales, solamente cuando las enfermedades analizadas se agruparon en las categorías de enfermedades parasitarias y enfermedades autoinmunes. Si se demostrara que las otras partes de esta hipótesis son ciertas, se podría sintetizar la información sobre plantas medicinales alrededor del mundo, para establecer así las plantas potencialmente más eficaces para llevarlas al laboratorio y analizarlas más profundamente.

French abstract

Par rapport aux recherches menées de façon aléatoire, les recherches effectuées par des critères ethnobotaniques et taxonomiques ont de meilleures chances à découvrir de nouvelles plantes médicinales à produit chimique actifs. Pendant les dernières décennies pourtant, l'ethnobotanique a réalisé peu de ces promesses à révéler un grand nombre de plantes médicinales et de nouveaux produits chimiques, testés au laboratoire. Avec les ressources limitées pour la recherche au laboratoire et le coût élevé des épreuves cliniques pour trouver de nouveaux candidats aux médicaments, il est difficile d'étudier, d'isoler et d'élucider la structure et le mécanisme des produits chimiques de chacune des nombreuses plantes médicinales (et les utilisations de ces plantes) décrites chaque année. Nous proposons une nouvelle technique théorique et quantitative pour préciser la recherche de nouvelles plantes médicinales; elle est basée sur l'hypothèse que les plantes étroitement apparentées, employées pour traiter les maladies étroitement apparentées dans les cultures très éloignées les unes des autres, ont une potentialité d'efficacité supérieure parce qu'elles représentent la découverte indépendante des propriétés chimiques semblables des plantes. Une partie de cette hypothèse-qui démontre que la sélection des plantes médicinales semblables est un choix rationnel et qu'il y a davantage de ressemblance dans la flore médicinale de deux cultures éloignées que dans leur flore générale-est examinée par un re-échantillonnage des données de recherches effectuées parmi les Malinké au Mali et les Asháninka au Pérou, en particulier sur la malaria, la maladie africaine du sommeil, la maladie de Chagas, la leishmania, le diabète, l'eczéma, l'asthme et les fibromes utérins. Dans ces cas précis, la similitude de la flore médicinale s'avère sensiblement plus grande que la similitude de la flore générale, mais seulement quand les maladies en question sont regroupées ensemble comme maladies parasitaires et auto-immunitaires. Si cette hypothèse est prouvée, elle permettra la synthèse des informations recueillies sur les plantes médicinales du monde entier pour en sélectionner de façon plus précise celles qui sont les plus efficaces et qui méritent analyse plus approfondie au laboratoire.

Asháninka abstract

Aayiantyarori iròpero aavintane, ontzimatye ancovacovatero ayotero ovaqueraripaye incashi iyoyetziri ashaninka, ayotzityaro aajatzi iyotane viracocha paitachari "quimica" ancantero aaca oshintsinka inchashipaye. Atziri yotacotzirori cametsa, ishtoriajacotzirori iyotane ashaninkapaye te iroñàrantero maaroni ocaratzi yamenacotaqueri laboratorioki. Aaviantyarori cametsa, ayotacotero aavintarontsiyetatsiri osamani antzimaventero ishtoriatacotaro, aajatzi osheki opinata ampinaventero aparopaye inchashi, acoviriqui ayotacotero, osaretsikipaye. Tzimatsi ovaquerari quenquishiriantsitatsiri ero opinata osheki ashitoriatacotero aparopaye inchashi, asampiyetatyrey pashinipaye atziri saicatsiri intaina puitarika inchasshi yavintari, ajatzirica oshiyaro ayotzi aaca, quemetachari atziri saikatsiri nampitsiki malinke aajatzi ishiyari ashaninka saicatsiri peruki, tzimatsi inchashi aajatzi yaavintari osheki okamètsatzi aririka anteri mantsiyarentsi icantaitziri ompetarentsi catsirentsi, pochokirentsi, patsarontsi(matatsi) ashipetate maaroni, ampochavathate, ancainikentsite, oncatsithakite tsinani. Aririka añaker aajatzi ahiyaro inchashi yaavintayetari pashinipaye atziri intainasatzi irdotake ahitoriatacoperoteri anàashityard aavintarontsi ovamairiri shithanentsi, onàshitaavintarontsi tzicaacoventairi ero antane mantsiyarentsi. Omanperotatyarica iròperotzi avintarontsi, oshitovake laboratorioki aritaque iyoitanaquero maaroni quipatsiki iroperori avintarontsi.

## Introduction

The field of ethnobotany is moving towards hypothesis-driven analytical research in recent years and away from simple inventories and descriptive work [1]. As part of this movement, quantitative ethnobotany is an increasingly useful field that is necessary both for analyzing the huge (and growing) amounts of plant use data being generated [2-5] and for improving the rigor and validity of ethnobotany as a science [6]. In general terms, Lewis et al. [7] have declared the success of ethnomedically-directed searches for new medicines from plants, stating a figure of 30% of the plant species collected using anti-infective ethnobotanical leads are found to have anti-HIV in *in vitro *tests (the hit rate), vs. only 8.5% for "random" screenings where every plant seen is collected for testing. The hit rate went up to 71.4% when plants used traditionally as antivirals were tested vs. the more general anti-infective category. They state the need for more of this type of research, including the search for more antimalarial plant compounds. The recent success with finding the anti-HIV drug prostratin in a Samoan medicinal plant *Homalanthus nutans *(G. Forst.) Guill. [Euphorbiaceae] [8] and the antidiarrheal compound crofelemer from the Western Amazonian plant *Croton lechleri *Müll. Arg. [Euphorbiaceae] [9] shows that there is hope for finding new medicines for epidemic diseases via ethnobotany while upholding indigenous intellectual property rights. McClatchey [10], however, explains that despite successes like prostratin modern bioprospecting from ethnomedical sources has largely failed and calls for better methods of analyzing and sharing of traditional medicinal plant knowledge. The goal of the "relational efficacy" quantitative technique describe here is to raise the hit rate above even the 30% seen with ethnobotanically-directed medicinal plant searches, i.e., to increase the efficiency of these searches.

Several promising techniques and conclusions have already arisen from quantitative ethnobotany: targeting medicinal plants for drug development that are in families with above average ratios of traditionally used medicinal species per total species in the family [4] by using residual values in a medicinal species vs. total species per family linear regression; showing how different cultures actually use rational (non-random and empirical) approaches in emphasizing certain taxa for their herbal remedies by focusing on plants with certain growth habits and ecology or in certain active families [4,11]; and using informant consensus– the number of healers who agree on a particular plant use- to corroborate the usefulness of certain plants and remove some uncertainty from collaborator interviews [6,12,3]. Albuquerque et al. [13] have shown how two indices, use values [3] and relative importance values, correlate when applied to the same data set, but diverge in certain cases because relative importance emphasizes the absolute number of uses and the use value emphasizes informant consensus. The relational efficacy index proposed here tries to combine these two approaches into one coherent measure by integrating cross-cultural and intra-cultural informant consensus as well as the disease-treating and plant-phylogenetic consensus.

Andrade-Cetto et al. [14] have introduced an interesting extension to informant consensus they call "disease consensus," which despite its name analyzes how multiple informants agree on and have knowledge of medicinal plants to treat one particular disease (not between several related diseases). This index tries to get around some of the inconsistencies of standard informant consensus techniques, but has yet to be definitively corroborated by other established indices or bioassays of disease treating efficacy. Reyes-García et al. [15] have compared eight common indices of traditional ecological knowledge and found them to correlate fairly well. Some of these indices can be independently validated (ecological cultural knowledge), giving some external validity to the other indices.

Johns et al. [16] has proposed a very interesting quantitative system for determining those plants in an ethnobotanical survey with the highest medical potential, based on a log-linear model that teases out what is called the "interaction effect," which is what is left when the higher likelihood of finding a common plant treatment for a common disease is controlled for in a matrix of plants and their medicinal uses. In other words, Johns et al. claim that this residual amount leftover when the probability of encountering common plants and common diseases is subtracted out explains the real efficacy of the plant medicine, a bit like Moerman's [4] residuals for medicinally speciose families. Although they call for verification by comparing the interaction-effect potential with efficacy determined by bioassays or current literature, they only attempt this qualitatively, not putting numbers on the medical efficacy of the plants found in the literature. Their work has been cited often e.g. [17-19], and the original authors have used this model in further studies [20,21], although they seem to reject the model for lack of statistical significance [22]. One shortcoming is that Johns et al. never defend their choice of a log-linear model to describe people's choices of medicinal plants. They also perform a sort of cross-cultural analysis with their results, noting that the top ten potential plants they have found are used similarly in many cultures, but again, the cross-cultural aspect of this analysis is not quantitative.

Browner et al. [23] have designed a system that allows quantitative cross-cultural comparisons of medicinal plant treatments by determining through biomedical literature searches which of the plants used by a certain culture for a particular disease have been shown to have some biochemical effect on the symptoms or causes of that disease. This is an enticing approach, combining a scientific and a cultural viewpoint while analyzing both a local cultural disorder, *susto*, and more physical female reproductive disorders, but their reliance on existing biomedical and biochemical literature means that rating and comparing plants that have not been studied in the laboratory is quite difficult. Juan et al. [24] have devised a quantitative method of finding similarities in traditional herbal medicine systems of Asia using statistical clustering algorithms on the plants used by each system to treat a set of diseases, but have stated that more innovative and broad methods are needed. Mace and Pagel [25] have formalized cross-cultural comparisons using methods borrowed from systematics, mapping out cultural traits such as plant use on language-based cultural phylogenies to determine if these traits are basal or derived. Ostraff [26] uses fuzzy clustering algorithms to look at how *tapa *cloth knowledge moves among several Polynesian islands. Weiss [27] shows how clustering algorithms can be used to find similarities in disease etiology and medicinal plants between the divergent traditional medicine of China and the Chatino of Mexico, elucidating some similarities in their concepts of disease causation.

Bennett and Prance [28] discuss related disease systems in deriving their species importance values from the number of body systems on which a medicinal plant species works and the number of pharmacological actions attributed to it, but this does not incorporate how these disease systems or actions are related. Yet the techniques described mainly allow only comparing and describing differences between cultures and their remedies, not the synthesis of several cultures' knowledge to pinpoint the potentially most-effective herbal remedies. Additionally, no one yet seems to have combined these methods of plant, disease, and cultural relatedness into one analytical system as proposed here.

## Plant knowledge communication

The ultimate goal of this research is to develop a set of formulas that will give us an estimate of the disease-treating potential of each plant species studied. Those plants with the highest potential would be the best candidates for undertaking the lengthy and expensive process of exploring their efficacy, phytochemistry and mechanisms of activity in the human body in the lab and in clinical trials, increasing the hit rate and lowering the cost of finding and testing new botanical medicines. This measure should be reproducible between different investigators and therefore objective and even useful in predicting the potential a certain species for which medicinal use data has not been collected may have for treating a certain uninvestigated disease.

One assumption of this technique is that the *less *related the cultures in the study are, such as Mali and Peru vs. Guatemala and Peru, the *less *chance those two cultures have had of communicating medicinal plant knowledge. If several unrelated cultures use closely related plants to treat the same disease, these discoveries of the effectiveness of the plants are *more *likely to be independent, and these plants should therefore be considered to have a *higher *potential than other plants that may be used for that disease in only one culture. To assess this assumption, the processes by which knowledge of medicinal plants is disseminated among cultures when different cultures interact and possibly intermingle needs to be well understood. Does the culture to which another culture migrates pick up a significant portion of the medicinal plant knowledge of the immigrant culture? Johnson [29], Palmer [30,31], Campos et al. [32] and Cox [33], have discussed these mechanisms of medicinal plant knowledge transfer, but this needs to be quantified on a more global basis. Lenaerts [34] confirms this concept for closely related cultures by showing that Peruvian Amazon indigenous groups like the Asháninka do not borrow medicinal plants based on the plants' efficacy from nearby groups such as the Shipibo, but rather based on each groups relations with and respect for their neighboring groups and their medicinal plant knowledge, with the caveat that the biomedical efficacies of the medicinal plants were not tested in the laboratory as part of this research.

The intercultural exchange of medicinal plants that do not undergo long-term experimentation in the culture that adopts these plants can confound the effects of experimentation that leads to acceptance of the most effective medicinal species. The ratio of medicinal species to total species in each plant family has been used in the past to make cross-cultural comparisons of medicinal plants, contrasting the medicinal flora of Jammu and Kashmir, India with that of the North American Indians [11] by comparing Moerman's [4] plant family residual values. Heinrich et al. [35] made some simple cross-cultural comparisons of Mexican indigenous groups and said that selection of plants in traditional medicine is definitely not random. Rather, a rational process of experimentation and exchanges between cultures goes on, sometimes up to a 70% exchange of medicinal plants with the example of the Gitksan of Western Canada and their neighboring groups [29].

Many of these studies have been ad hoc, asking only whether the two cultures are connected or not, instead of how connected they are, losing some of the information in their analysis of the measure of relatedness of cultures. There is an important quantitative difference between two neighboring groups in Peru using similar plants to treat a disease, and groups in Peru and Mali using similar plants to treat the same disease. The latter case is much more suggestive that the two cultures independently discovered similar plant uses, and that this was not communicated plant knowledge as in the example of the Gitksan [29]. Campos and Ehringhaus [32] have found that a quarter to a third of species-specific plant uses of two indigenous groups in the Brazilian Amazon, the Kaxinawá and Yawanawá, have been acquired from neighboring non-indigenous *seringueiros *(rubber tappers) or *ribeirinhos *(river dwelling people). Cox [33] claims that much of Polynesian herbal medicine is an indigenous tradition although there are some introductions, and that 66% of medicinal plants used in Polynesia are not used elsewhere, and are therefore unlikely to be European introductions, while 34% have some use outside of Polynesia. In various studies analyzed between 1838 and 2002, Palmer [30] found that anywhere from 14–53% of medicinal plants used in Hawai'i were Polynesian introduced species, although this is a bit different from introduced *uses*. These figures contrast Johnson's much higher 70% shared medicinal plant use figure, perhaps because of the greater cultural and geographic proximity of the Gitksan and their neighbors. If this degree of relations of the cultures being studies can be quantified as I am proposing, it can give us much more information about how much medicinal plant knowledge the cultures would naturally share.

The possible explanations for two different cultures using similar plants to treat related diseases are:

1. The two cultures have independently discovered that these two related plants treat the diseases effectively through experimentation and have not communicated these uses to each other. This explanation best fits the stated theory.

2. The two cultures have independently decided to use these two related plants to treat the diseases, but one or both of the cultures has used the plants only for a short time, without much experimentation, and therefore there is less evidence that these plants are medicinally effective.

3. The two cultures have independently decided to use these two related plants to treat the diseases through the doctrine of signatures, which is a common method of medicinal plant discovery around the world [36], and the related diseases are likely to effect the same organ system and the related plants are likely to look the same.

4. The two cultures have communicated to each other this medicinal plant use through immigration, literature, or other media moving from one culture to another.

The reason that it is important to look at less related cultures is that with increasing distance between cultures, the probability of option 4 goes down and the probabilities of options 1, 2, and 3 increase, with less possibility of communication. The ratio of options 1, 2, and 3 to each other is unclear, but asking questions such as how long a medicinal plant has been used during interviews helps to increase the probability of option 1 vs. options 2 and 3, as there has been more time for experimentation and verification with a particular plant remedy. Using informant consensus techniques during interviews about a medicinal plant [3] can act as a stand-in for the length of use of the plant remedy as a higher informant consensus value indicates that the plant has been better tested by the community, again increasing the probability of option 1.

Giving many clear examples, Bennett [36] proposes that the doctrine of signatures is a mnemonic method for remembering many medicinal plants, rather than a method of choosing medicinal plants merely based on their signatures. This implies that plants to which the doctrine of signature applies are actually quite well tested and known to be effective, rather than being chosen merely because they resemble the disease or affected organ. Accepting this conclusion would lead to option 3 being less of a confounding factor, as the plants would be well tested as in option 1.

## A comparative case study using families and genera

The two distant cultures of the Asháninka of Peru and the Malinké of Mali and the seven diseases malaria, African sleeping sickness (trypanosomiasis), Chagas disease, leishmaniasis, asthma, eczema, diabetes, and uterine fibroids were selected to test the prerequisite hypothesis to the general theory that the medicinal flora of two distant cultures are significantly more similar to each other than the general flora of the two cultures areas are similar.

### Cultures

In choosing cultures for this study, the more remote and more recently contacted a group is the better, as they will have less chance of introduced plant uses. Although the cultures selected must be as distant as possible, it is also necessary that they share some elements of their floras. The areas compared need not have the exact same species, but if they share some genera or families it will make determining the plants' relatedness values easier. In comparing the flora of Peruvian Amazon and the dry savannas of Mali, we have found that 21% of their genera overall and 30% of the medicinal plant genera of the Mali savannas are also found in Southwest Amazon area of Peru [37-40]. So although at first glance it might seem ludicrous to try to compare the medicinal floras of such divergent habitats as a rainforest and a savanna, this flora overlap percentage is high enough to make a more in-depth comparison of the medicinal plants of the two areas. The fact that the cultures of the Peruvian Amazon and the Malian savannas are so distantly related that they are very unlikely to have communicated medicinal plant uses to each other also raises the probability that any related plants used by both of them to treat related diseases are independent discoveries, which strengthens the quantitative model. Lewis et al. [23] has suggested the same idea that use of similar medicinal plants by nearby Jívaro communities in the Peruvian Amazon corroborates those uses and the medicinal efficacy of the plants.

The Asháninka (alternatively Campa, Asháninca, Ashéninka, and Ashéninca), the fourth largest indigenous group in Peru after the Quechua, Aymara, and Aguaruna, number about 25,000–30,000, and speak the Asháninka language in the Arawak language family, which is divided into 4 dialects: Pajonal, Yuruá-Ucayali, Perené, and Pichis [41,42]. Spread throughout southern Amazonian Peru and extending into neighboring Acre, Brazil, the Asháninka enjoy immense notoriety for introducing the antirheumatic use of their medicinal plant cat's claw or *uña de gato *(*Uncaria tomentosa *(Willd. ex Roem. & Schult.) DC. and *U. guianensis *(Aubl.) J.F. Gmel. [Rubiaceae]) [43] to the world, yet there is a paucity of ethnobotanical data on them. This plant is now used throughout Peru and much of the Western world for arthritis, asthma, cancer, contraception, fevers, ulcers, wound healing, and urinary tract inflammations, to name a few uses, but Keplinger claims to have proven its effectiveness as an immune booster and is currently working on marketing a drug in Europe for rheumatoid arthritis derived from *U. tomentosa *called Saventaro™, the Asháninka name for this plant. There is much written about the Asháninka in general [44-49], about their political situation stemming from conflict with the Shining Path revolutionaries in Peru [50-52], linguistic and cultural anthropology [42,53-56], and there is some recent ethnobotanical work on the Asháninka food plants, medicinal plants and medical system [57]. Because of the worldwide acclaim and use of cat's claw introduced by the Asháninka, the rest of their herbal pharmacopoeia deserves study. As Lenaerts [57] has described, the Asháninka medical system emphasizes the relations of people, plants, and diseases making them a perfect fit for the theoretical "relational efficacy" system.

**Figure 1:**
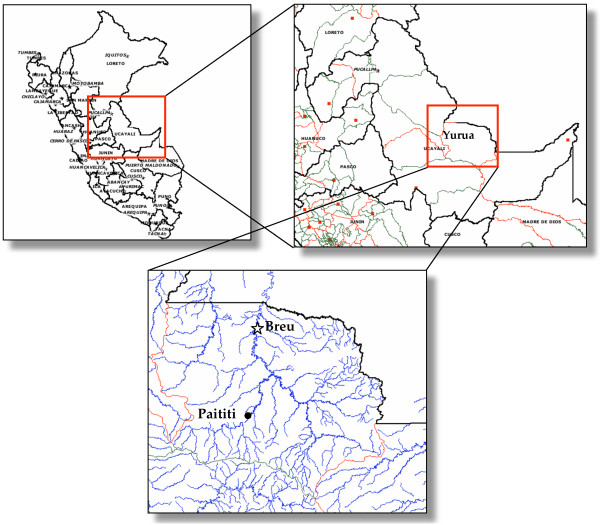
**Paititi, Peru**. The Peruvian study site Paititi is in the Amazonian district of Yuruá in the department (state) of Ucayali, along the Huacapishtea River.

The Asháninka community of Paititi is located in the Southwest Amazon vegetation zone in the Ucayali Department of Peru, near the Brazilian border (see Figure 1). The Asháninka who live in Paititi mostly speak the Yuruá Asháninka dialect, although some speak the Perené dialect as well, and there are one to two visiting teachers who are indigenous Shipibo, also from Ucayali Department. In the two years of fieldwork in Paititi (2003 and 2004) the population of the community fluctuated between 25–30 people, comprising 6 families living in separate palm thatch and wood houses. The surrounding agricultural fields and rainforest are typical of the Southwest Amazon habitat [58].

In Mali, working with the *Département de Médecine Traditionnelle *(Department of Traditional Medicine, DMT) in the capital Bamako, and their connections with the *Association des Thérapeutes Traditionnels de Kita *(Association of Traditional Healers of Kita) in Kita, in the Western extent of Mali, I was able to interview fifteen Malinké healers during field work in 2004. The Malinké, one of the largest ethnic groups in Mali, with about 600,000 members, speak a combination of French, Bamanakan, and Malinké, and are generally Muslim, animist, or a combination thereof [41,59]. There is little ethnobotanical work solely on the Malinké, mostly because they are intermixed with the Songhay, Pelou, Bozo, Tuareg and other ethnic groups throughout Mali, but many of their medicinal plants are included in works on the ethnobotany of West Africa [37,38,60,61].

**Figure 2:**
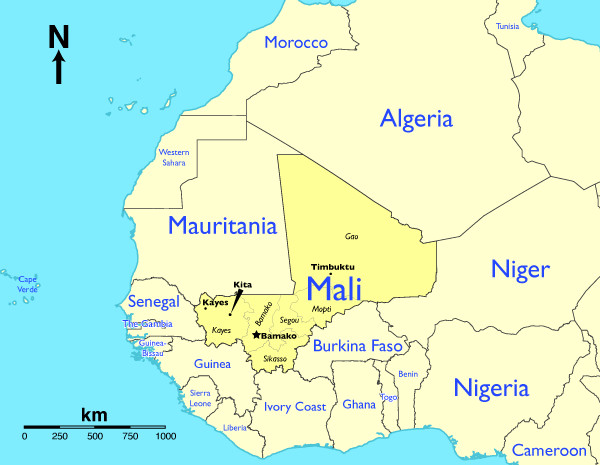
**Kita, Mali**. The Malian field site Kita is located in the west of Mali, in West Africa, in savanna vegetation.

The field site of Kita, in the western end of Mali (see Figure 2), is in the Sudanese savanna area with some Guinean gallery forest vegetation type reaching up into the southern end of the town but with fewer of the baobab trees (*Adansonia digitata *L. [Malvaceae]) common in the eastern part of the country [61].

### Diseases

A criterion for selecting diseases to study is to find diseases that occur in Peru and/or Mali and are related and have the same underlying cause in the body. Using these criteria, malaria, African sleeping sickness, Chagas' disease, leishmaniasis, diabetes, eczema, asthma, and uterine fibroids have been selected. Diabetes, eczema, and asthma were picked as all three are autoimmune diseases, with the latter two more closely associated in the "auto-immune triad". The third member of this autoimmune triad is hay fever, which was not included in this study because it is not thought to be common in the indigenous groups selected. If one culture treats asthma with a certain plant and another distant culture treats eczema with the same plant, although these diseases seem superficially very different, they are considered closely related auto-immune diseases by Western medicine and therefore could be treated by the same plant chemicals acting on the underlying mechanism of the immune system. Thus, these two distant uses of the same plant for eczema and asthma can be considered similar uses, raising the estimate of the efficacy of this plant.

Malaria, leishmaniasis, African sleeping sickness, and Chagas' disease are all caused by a protozoan parasite infection, the latter three more specifically by a trypanosome (family Trypanosomatidae), and the latter two being in the same genus *Trypanosoma *[62-64], thereby exhibiting different degrees of evolutionary proximity. Studying uterine fibroids allows comparison of my work in Peru with ethnobotanical research that has been done on this disease in Chile and among Dominican and Chinese groups in New York City by the Rosenthal Center for Complementary and Alternative Medicine at the Columbia-Presbyterian Medical Center (CPMC) [65]. Of the diseases mentioned, however, uterine fibroids is the most difficult to study solely with interviews as it has few outwardly apparent symptoms.

### Methodology

To accomplish this cross-cultural study, ethnobotanical data was gathered in structured interviews and plant collections with healers of the indigenous Asháninka of Paititi village in Ucayali, Peru and the Malinké of Kita, Mali in 2003 and 2004, focusing on plants used to treat malaria, African sleeping sickness, Chagas' disease, leishmaniasis, asthma, eczema, diabetes, and uterine fibroids. Prior informed consent forms cleared with the City University of New York Institutional Review Board (CUNY IRB) on human subjects were signed with everyone interviewed that guaranteed immediate compensation for the healer's time, return of documentation of the results of the study to the community [66], that pharmaceuticals would not made from the medicinal plants described in the study, and that the names of the plants would not be revealed to anyone outside the study.

Cultural notions of disease are difficult to deal with because of different symptomatic descriptions for what may be the same underlying disease. Working with medical texts, doctors, translators, and healers of each culture, the symptoms of a disease in that culture and the name of the disease in the local language (Asháninka or Bambara) were determined to help resolve this issue. During interviews, diseases were at first only described by their Western medical symptoms, not by name, and the collaborator was asked to name the disease and the plants used to treat it. If a particular collaborator did not give the name of the disease in their language based the stated symptoms, they would be interviewed again later about the same disease, but the second time it would be named in their local language and in the country's official language (Spanish or French) if they spoke that language. This dual description of each disease by symptoms and name will provide valuable information on whether more effective medicinal plants are found by describing symptoms or by naming diseases, once the efficacy of each plant has been determined.

With the help of the collaborators, species described in the interviews were collected in quadruplicate when accessible to make into a small-scale reference herbarium for the communities in the study; for deposit in the study countries' main herbaria, Universidad Nacional Mayor de San Marcos (USM) in Peru and the Département de Médecine Traditionnelle in Mali (not listed in Index Herbariorum); for sending to a family expert at other herbaria; and for deposit in my institutional herbarium (NY). Species were identified with the help of Gentry [67], Arbonnier [61], the aforementioned herbaria's collection, their staff, and several taxonomic experts.

The families and genera of the general flora have been determined for Kita, Mali from Arbonnier [61] and for Paititi, Peru from Daly and Silvera [58] which covers the state of Acre, Brazil, which is also in the Southwest Amazon floristic zone in which Paititi is found. However, because of the prior informed consent agreements with my collaborators, neither species, genus, nor family names are given, as has become fairly common practice in recent medical ethnobotanical research [68,5,73]. This paper shows that despite not revealing plant names, there is interesting work that can be published with this data that advances the science of ethnobotany. A summary of the field collections and overlap percentages (the number of each taxa that were found in both field sites divided by the number of total taxa found when the taxa from both field sites are combined, or intersection of the taxa/union of the taxa) is given in Table 1.

**Table 1:** A summary of the collections of the medicinal plants of the Asháninka of Peru and the Malinké of Mali, and their overlap percentages.

	**Collections**	**Species**	**Genera**	**Families**
Peru	86	74	73	39
Mali	90	80	54	41
Both	n/a	3	11	25

Total	176	152	116	55
Overlap (Both/Total)		2%	10%	46%

### Analysis

These original data comprise an accurate database of shared plant uses which can be analyzed using the described quantitative system and compared with data gathered on the same diseases from other areas of the world. Further literature research on the collected plant species and chemical analysis will be necessary to measure the relatedness of the plants, cultures, and diseases involved in the study using dated phylogenies and to calibrate the quantitative system using well-studied medicinal plants.

Part of the hypothesis of this work is that the medicinal floras of the different cultures are taxonomically more similar than the general floras of the geographic areas where the cultures are located. This hypothesis is relatively easy to test using contingency tables of the overlapping medicinal and general flora of the two cultures, the odds-ratio or Jaccard similarity index of these tables, and resampling statistic techniques, a technique that recalculates statistics thousands of times while resampling from collected data [74]. Approximate randomization or resampling statistics techniques are in the same family of numerical approaches to statistical analysis that sample without replacement as Monte Carlo methods, essentially reshuffling the labels or experimental group on each collected datum. Monte Carlo methods differ, however, in that they create new data based on theoretical probability distributions of the system under study.

Contingency tables are used in statistical comparisons of counts of occurrences of outcomes in several populations with different experimental groups, most often in two by two tables. The odds ratio (OR) statistic is calculated as

$$\frac{N_{present\;in\;Peru\text{, }present\;in\;Mali}N_{absent\;in\;Peru\text{, }absent\;in\;Mali}}{N_{present\;in\;Peru\text{, }absent\;in\;Mali}N_{absent\;in\;Peru\text{, }present\;in\;Mali}}$$

where each *N *is the count from one of the four central squares of the contingency table, which would be

$$\frac{57\cdot 266}{136\cdot 6}=18.581$$

in the case of Table 2, comparing the families of the general flora of Peru and Mali. In these tables, the OR explains that an outcome is a certain amount more likely for one experimental group versus another, e.g. that if a family is present in the Peruvian Amazon flora it is 18.581 times more likely to be present than absent in the Malian savanna flora in the case shown in Table 2.

**Table 2:** The overlap of general and medicinal families and genera found in the Southwest Amazon area of Peru and in Mali, as contingency tables, with the significance of each table, and for all but Tables 2 and 3 (the general flora comparison), whether the odds ratio and Jaccard similarity is significantly greater than the odds ratio and Jaccard similarity for the general flora. Odds ratios and Jaccard similarities that are greater than the corresponding values for the general flora (Tables 2 and 3) are italicized.

***Families***					
**General flora family comparison**					
		Peru			
		present	absent		
Mali	present	57	6	63	
	absent	136	266	402	
		193	272	465	
	Odds ratio:	18.581		p value:	1.6849 × 10^-18^
	Jaccard similarity:	0.286			

The Jaccard similarity index is a measure of the overlap of the two sets and is calculated by the intersection of the two sets divided by the union of the two sets, i.e.

$$\frac{N_{present\;in\;Peru\text{, }present\;in\;Mali}}{N_{present\;in\;Peru\text{, }present\;in\;Mali}+N_{present\;in\;Peru\text{, }absent\;in\;Mali}+N_{absent\;in\;Peru\text{, }present\;in\;Mali}}$$

It should be noted that the Jaccard similarity calculation does not use the number of taxa absent from both sets (e.g. 266 in Table 2), while the odds ratio calculation does.

The null hypothesis H_o _here that we wish to reject is that just by chance the two cultures have wound up with similar medicinal floras merely by selecting from similar general floras, i.e., the odds-ratio or Jaccard similarity index of the medicinal flora is no greater than the odds-ratio or Jaccard index of the general flora than chance would allow. Resampling statistics will here allow the calculation of the statistical significance of the difference in the similarity of the medicinal and general taxa contingency tables, a significance whose calculation is not well defined using standard exact statistical techniques, by reshuffling the numbers in the two contingency tables' categories thousands of times, keeping the row totals the same and recalculating the similarity difference between the tables for each reshuffle. The significance p then is computed as N_g_/N_t _where N_g _is the number of reshuffles where the medicinal floras' similarity is higher than the similarity of the general floras and N_t _is the total number of reshuffles.

To calculate these similarities, contingency tables were created of the families and genera found in the medicinal and general flora of the Southwest Amazon area of Peru and in Mali, using Angiosperm Phylogeny Group [75] designations and total worldwide counts for families and genera. Resampling statistics were calculated 10,000 times using a 500 MHz Apple PowerBook Pismo running Resampling Stats version 4.0 [76], with each table comparison run taking several minutes to complete. There resulting list of contingency tables, odds-ratios, similarity values, and significance for families and genera for each disease and disease category as well as the general flora are given in Tables 2, 3, 4, 5, 6, 7, 8, 9, 10, 11, 12, 13, 14, 15, 16, 17, 18, 19, 20, 21.

**Table 3:** The overlap of general and medicinal families and genera found in the Southwest Amazon area of Peru and in Mali, as contingency tables, with the significance of each table, and for all but Tables 2 and 3 (the general flora comparison), whether the odds ratio and Jaccard similarity is significantly greater than the odds ratio and Jaccard similarity for the general flora. Odds ratios and Jaccard similarities that are greater than the corresponding values for the general flora (Tables 2 and 3) are italicized.

***Genera***					
**General flora genus comparison**					
		Peru			
		present	absent		
Mali	present	93	132	225	
	absent	1045	11996	13041	
		1138	12128	13266	
	Odds ratio:	8.088		p value:	3.2759 × 10^-41^
	Jaccard similarity:	0.073			

**Table 4:** The overlap of general and medicinal families and genera found in the Southwest Amazon area of Peru and in Mali, as contingency tables, with the significance of each table and whether the odds ratio and Jaccard similarity is significantly greater than the odds ratio and Jaccard similarity for the general flora. Odds ratios and Jaccard similarities that are greater than the corresponding values for the general flora (Tables 2 and 3) are italicized.

**All medicinal family comparison**					
		Peru			
		present	absent		
Mali	present	25	14	39	
	absent	16	410	426	
		41	424	465	
	Odds ratio:	*45.759*		p value:	2.291 × 10^-21^
				p value (OR > general OR):	0.057994
	Jaccard similarity:	*0.455*		p (Jaccard > general):	0.0001

**Table 5:** The overlap of general and medicinal families and genera found in the Southwest Amazon area of Peru and in Mali, as contingency tables, with the significance of each table and whether the odds ratio and Jaccard similarity is significantly greater than the odds ratio and Jaccard similarity for the general flora. Odds ratios and Jaccard similarities that are greater than the corresponding values for the general flora (Tables 2 and 3) are italicized.

**All medicinal genus comparison**					
		Peru			
		present	absent		
Mali	present	11	43	54	
	absent	62	13150	13212	
		73	13193	13266	
	Odds ratio:	*54.257*		p value:	3.4275 × 10^-15^
				p value (OR > general OR):	0.027597
	Jaccard similarity:	*0.095*		p (Jaccard > general):	0.0001

**Table 6:** The overlap of general and medicinal families and genera found in the Southwest Amazon area of Peru and in Mali, as contingency tables, with the significance of each table and whether the odds ratio and Jaccard similarity is significantly greater than the odds ratio and Jaccard similarity for the general flora. Odds ratios and Jaccard similarities that are greater than the corresponding values for the general flora (Tables 2 and 3) are italicized.

**Parasitic family comparison**					
		Peru			
		present	absent		
Mali	present	13	10	23	
	absent	11	431	442	
		24	441	465	
	Odds ratio:	*50.936*		p value:	1.5238 × 10^-13^
				p value (OR > general OR):	0.091491
	Jaccard similarity:	*0.382*		p (Jaccard > general):	0.0001

**Table 7:** The overlap of general and medicinal families and genera found in the Southwest Amazon area of Peru and in Mali, as contingency tables, with the significance of each table and whether the odds ratio and Jaccard similarity is significantly greater than the odds ratio and Jaccard similarity for the general flora. Odds ratios and Jaccard similarities that are greater than the corresponding values for the general flora (Tables 2 and 3) are italicized.

**Parasitic genus comparison**					
		Peru			
		present	absent		
Mali	present	7	31	38	
	absent	20	13208	13228	
		27	13239	13266	
	Odds ratio:	*149.123*		p value:	4.2180 × 10^-13^
				p value (OR > general OR):	0.011299
	Jaccard similarity:	*0.121*		p (Jaccard > general):	0.0001

**Table 8:** The overlap of general and medicinal families and genera found in the Southwest Amazon area of Peru and in Mali, as contingency tables, with the significance of each table and whether the odds ratio and Jaccard similarity is significantly greater than the odds ratio and Jaccard similarity for the general flora. Odds ratios and Jaccard similarities that are greater than the corresponding values for the general flora (Tables 2 and 3) are italicized.

**Trypanosomal family comparison**					
		Peru			
		present	absent		
Mali	present	7	6	13	
	absent	8	444	452	
		15	450	465	
	Odds ratio:	*64.750*		p value:	5.0237 × 10^-09^
				p value (OR > general OR):	0.10409
	Jaccard similarity:	*0.333*		p (Jaccard > general):	0.0001

**Table 9:** The overlap of general and medicinal families and genera found in the Southwest Amazon area of Peru and in Mali, as contingency tables, with the significance of each table and whether the odds ratio and Jaccard similarity is significantly greater than the odds ratio and Jaccard similarity for the general flora. Odds ratios and Jaccard similarities that are greater than the corresponding values for the general flora (Tables 2 and 3) are italicized.

**Trypanosomal genus comparison**					
		Peru			
		present	absent		
Mali	present	3	12	15	
	absent	15	13236	13251	
		18	13248	13266	
	Odds ratio:	*220.600*		p value:	5.2761 × 10^-07^
				p value (OR > general OR):	0.018798
	Jaccard similarity:	*0.100*		p (Jaccard > general):	0.0001

**Table 10:** The overlap of general and medicinal families and genera found in the Southwest Amazon area of Peru and in Mali, as contingency tables, with the significance of each table, and whether the odds ratio and Jaccard similarity is significantly greater than the odds ratio and Jaccard similarity for the general flora. Odds ratios and Jaccard similarities that are greater than the corresponding values for the general flora (Tables 2 and 3) are italicized.

**Malaria family comparison**					
		Peru			
		present	absent		
Mali	present	4	15	19	
	absent	5	441	446	
		9	456	465	
	Odds ratio:	*23.520*		p value:	0.00014609
				p value (OR > general OR):	0.31347
	Jaccard similarity:	0.167		p (Jaccard > general):	1

**Table 11:** The overlap of general and medicinal families and genera found in the Southwest Amazon area of Peru and in Mali, as contingency tables, with the significance of each table and for all but Tables 2 and 3 whether the odds ratio and Jaccard similarity is significantly greater than the odds ratio and Jaccard similarity for the general flora. Odds ratios and Jaccard similarities that are greater than the corresponding values for the general flora (Tables 2 and 3) are italicized.

**Malaria genus comparison**					
		Peru			
		present	absent		
Mali	present	2	30	32	
	absent	7	13227	13234	
		9	13257	13266	
	Odds ratio:	*125.971*		p value:	0.00012384
				p value (OR > general OR):	0.022198
	Jaccard similarity:	0.051		p (Jaccard > general):	1

**Table 12:** The overlap of general and medicinal families and genera found in the Southwest Amazon area of Peru and in Mali, as contingency tables, with the significance of each table and whether the odds ratio and Jaccard similarity is significantly greater than the odds ratio and Jaccard similarity for the general flora. Odds ratios and Jaccard similarities that are greater than the corresponding values for the general flora (Tables 2 and 3) are italicized.

**Autoimmune family comparison**					
		Peru			
		present	absent		
Mali	present	13	13	26	
	absent	15	424	439	
		28	437	465	
	Odds ratio:	*28.267*		p value:	2.1699 × 10^-11^
				p value (OR > general OR):	0.27887
	Jaccard similarity:	*0.317*		p (Jaccard > general):	0.0001

**Table 13:** The overlap of general and medicinal families and genera found in the Southwest Amazon area of Peru and in Mali, as contingency tables, with the significance of each table and whether the odds ratio and Jaccard similarity is significantly greater than the odds ratio and Jaccard similarity for the general flora. Odds ratios and Jaccard similarities that are greater than the corresponding values for the general flora (Tables 2 and 3) are italicized.

**Autoimmune genus comparison**					
		Peru			
		present	absent		
Mali	present	4	36	40	
	absent	35	13191	13226	
		39	13227	13266	
	Odds ratio:	*41.876*		p value:	4.0460 × 10^-06^
				p value (OR > general OR):	0.10629
	Jaccard similarity:	0.053		p (Jaccard > general):	1

**Table 14:** The overlap of general and medicinal families and genera found in the Southwest Amazon area of Peru and in Mali, as contingency tables, with the significance of each table and whether the odds ratio and Jaccard similarity is significantly greater than the odds ratio and Jaccard similarity for the general flora. Odds ratios and Jaccard similarities that are greater than the corresponding values for the general flora (Tables 2 and 3) are italicized.

**Eczema family comparison**					
		Peru			
		present	absent		
Mali	present	4	13	17	
	absent	5	443	448	
		9	456	465	
	Odds ratio:	*27.262*		p value:	8.7749 × 10^-05^
				p value (OR > general OR):	0.28537
	Jaccard similarity:	0.182		p (Jaccard > general):	1

**Table 15:** The overlap of general and medicinal families and genera found in the Southwest Amazon area of Peru and in Mali, as contingency tables, with the significance of each table and whether the odds ratio and Jaccard similarity is significantly greater than the odds ratio and Jaccard similarity for the general flora. Odds ratios and Jaccard similarities that are greater than the corresponding values for the general flora (Tables 2 and 3) are italicized.

**Eczema genus comparison**					
		Peru			
		present	absent		
Mali	present	1	19	20	
	absent	9	13237	13246	
		10	13256	13266	
	Odds ratio:	*77.409*		p value:	0.010375
				p value (OR > general OR):	0.014399
	Jaccard similarity:	0.034		p (Jaccard > general):	1

**Table 16:** The overlap of general and medicinal families and genera found in the Southwest Amazon area of Peru and in Mali, as contingency tables, with the significance of each table and whether the odds ratio and Jaccard similarity is significantly greater than the odds ratio and Jaccard similarity for the general flora. Odds ratios and Jaccard similarities that are greater than the corresponding values for the general flora (Tables 2 and 3) are italicized.

**Diabetes family comparison**					
		Peru			
		present	absent		
Mali	present	2	13	15	
	absent	17	433	450	
		19	446	465	
	Odds ratio:	3.919		p value:	0.13742
				p value (OR > general OR):	1
	Jaccard similarity:	0.063		p (Jaccard > general):	1

**Table 17:** The overlap of general and medicinal families and genera found in the Southwest Amazon area of Peru and in Mali, as contingency tables, with the significance of each table and whether the odds ratio and Jaccard similarity is significantly greater than the odds ratio and Jaccard similarity for the general flora. Odds ratios and Jaccard similarities that are greater than the corresponding values for the general flora (Tables 2 and 3) are italicized.

**Diabetes genus comparison**					
		Peru			
		present	absent		
Mali	present	0	15	15	
	absent	22	13229	13251	
		22	13244	13266	
	Odds ratio:	0.000		p value:	1
				p value (OR > general OR):	1
	Jaccard similarity:	0.000		p (Jaccard > general):	1

**Table 18:** The overlap of general and medicinal families and genera found in the Southwest Amazon area of Peru and in Mali, as contingency tables, with the significance of each table and whether the odds ratio and Jaccard similarity is significantly greater than the odds ratio and Jaccard similarity for the general flora. Odds ratios and Jaccard similarities that are greater than the corresponding values for the general flora (Tables 2 and 3) are italicized.

**Asthma family comparison**					
		Peru			
		present	absent		
Mali	present	4	9	13	
	absent	8	444	452	
		12	453	465	
	Odds ratio:	*24.667*		p value:	0.00010722
				p value (OR > general OR):	0.29017
	Jaccard similarity:	0.190		p (Jaccard > general):	1

**Table 19:** The overlap of general and medicinal families and genera found in the Southwest Amazon area of Peru and in Mali, as contingency tables, with the significance of each table and whether the odds ratio and Jaccard similarity is significantly greater than the odds ratio and Jaccard similarity for the general flora. Odds ratios and Jaccard similarities that are greater than the corresponding values for the general flora (Tables 2 and 3) are italicized.

**Asthma genus comparison**					
		Peru			
		present	absent		
Mali	present	1	16	17	
	absent	11	13238	13249	
		12	13254	13266	
	Odds ratio:	*75.216*		p value:	0.010655
				p value (OR > general OR):	0.014599
	Jaccard similarity:	0.036		p (Jaccard > general):	1

**Table 20:** The overlap of general and medicinal families and genera found in the Southwest Amazon area of Peru and in Mali, as contingency tables, with the significance of each table and whether the odds ratio and Jaccard similarity is significantly greater than the odds ratio and Jaccard similarity for the general flora. Odds ratios and Jaccard similarities that are greater than the corresponding values for the general flora (Tables 2 and 3) are italicized.

**Fibroids family comparison**					
		Peru			
		present	absent		
Mali	present	6	14	20	
	absent	6	439	445	
		12	453	465	
	Odds ratio:	*31.357*		p value:	1.2956 × 10^-06^
				p value (OR > general OR):	0.33487
	Jaccard similarity:	0.231		p (Jaccard > general):	1

**Table 21:** The overlap of general and medicinal families and genera found in the Southwest Amazon area of Peru and in Mali, as contingency tables, with the significance of each table and whether the odds ratio and Jaccard similarity is significantly greater than the odds ratio and Jaccard similarity for the general flora. Odds ratios and Jaccard similarities that are greater than the corresponding values for the general flora (Tables 2 and 3) are italicized.

**Fibroids genus comparison**					
		Peru			
		present	absent		
Mali	present	3	23	26	
	absent	10	13230	13240	
		13	13253	13266	
	Odds ratio:	*172.565*		p value:	1.0923 × 10^-06^
				p value (OR > general OR):	0.024098
	Jaccard similarity:	*0.083*		p (Jaccard > general):	0.0001

When these contingency tables comparing the general flora of Mali and the Southwest Amazon area of Peru with the medicinal plants of the Asháninka and Malinké are examined, it is clear that there is significant similarity within the general flora and the medicinal flora from the G test, and that the medicinal flora has a significantly higher similarity between the two areas than the general flora's similarity. It can be seen from these tables that in all cases where the medicinal flora similarity or odds-ratio is greater than those of the general flora (numbers in italics), it is statistically significant. This allows us to accept our prerequisite hypothesis H_1_, but if we look more deeply into the disease categories and the difference between the genus and family taxa levels, the results become more complicated and less consistent. There seems to be more significant results of higher similarity in medicinal plants for individual diseases and categories than in the general flora when looking at genera rather than families, as shown in Tables 2, 3, 4, 5, 6, 7, 8, 9, 10, 11, 12, 13, 14, 15, 16, 17, 18, 19, 20, 21 and the summary in Table 22. There is also variation in significance when looking at different disease ranks, i.e., individual disease vs. disease categories such as parasitic or autoimmune diseases.

**Table 22:** A summary of the significance of the the Jaccard similarity (Sim.) and odds ratio (OR) for the families and genera of plants used to treat each disease category between the Asháninka and Malinké being higher than the general flora similarity and odds ratio between the Asháninka and Malinké (○: p < 0.1, ●: p < 0.05, ●●●●: p < 0.0001, NS: not significant, p > 0.1)

	**Families**		**Genera**	
**Diseases**	**Sim.**	**OR**	**Sim.**	**OR**
All studied	●●●●	○	●●●●	●
Parasitic	●●●●	○	●●●●	●
Trypanosomal	●●●●	NS	●●●●	●
Malaria	NS	NS	NS	●
Auto-immune	●●●●	NS	NS	NS
Eczema	NS	NS	NS	●
Diabetes	NS	NS	NS	NS
Asthma	NS	NS	NS	●
Uterine fibroids	NS	NS	●●●●	●

Table 23 shows the distribution of plant taxa used to treat different diseases and categories in Peru, Mali, and the combination thereof. In the combined medicinal flora of Peru and Mali the majority of plant taxa are used to treat autoimmune diseases (20.60% of families present, 5.91% of genera present), and within in this category, diabetes has the highest representation (16.08% of families present, 3.23% of genera present). Parasitic diseases are the second-highest-represented category (17.59% of families present, 4.57% of genera present), with malaria best represented within this category (20.60% of families present, 5.91% of genera present). For the Peruvian medicinal flora, this same pattern of the predominance of autoimmune diseases and diabetes within that continues, but within the second-highest-represented category of parasitic diseases, leishmaniasis predominates rather than malaria (5.70% of families present, 1.14% of genera present). This may be due to leishmaniasis being native to South America or at least present in South America much longer than malaria, a relatively recent introduction [77]. In Mali, auto-immune diseases are still the predominant category (41.27% of families present, 17.78% of genera present), but within this category, eczema rather than diabetes is the best represented (5.70% of families present, 1.14% of genera present), most likely because of the drier environmental conditions that often bring on eczema and fewer of the high-starch foods such as yuca (*Manihot esculenta *Crantz [Euphorbiaceae]) that can exacerbate diabetes. Within the parasitic disease category in Mali, also the second best represented, malaria is best represented (30.16% of families present, 14.22% of genera present) as opposed to leishmaniasis in Peru, most likely due to malaria's origins in Africa [78]. It should be noted that these differences are only statistically significant when considering the combined medicinal floras of Peru and Mali and the disease categories, not individual diseases.

**Table 23:** The distribution of plant families and genera used treat each disease and disease category in Peru, Mali, and the combined medicinal flora of the two areas. Those distributions with a significant difference based on a log-likelihood ratio test (G-test) are marked with a footnote, all other pairwise comparisons of categories or diseases are not significantly different.

	**Mali**				**Peru**				**Combined**			
	**Families**		**Genera**		**Families**		**Genera**		**Families**		**Genera**	
**Disease**	**n**	**percent of present**	**n**	**percent of present**	**n**	**percent of present**	**n**	**percent of present**	**n**	**percent of present**	**n**	**percent of present**
Parasitic	23	36.51%	38	16.89%	24	12.44%	27	2.37%	35^b^	17.59%	58^cd^	4.57%
Malaria	19	30.16%	32	14.22%	9	4.66%	9	0.79%	25	12.56%	39	3.07%
Trypanosomes^a^	13	20.63%	15	6.67%	15	7.77%	18	1.58%	22	11.06%	30	2.36%
Chagas	0	0.00%	0	0.00%	4	2.07%	5	0.44%	4	2.01%	13	1.02%
Leishmaniasis	0	0.00%	0	0.00%	11	5.70%	13	1.14%	11	5.53%	5	0.39%
Autoimmune	26	41.27%	40	17.78%	28	14.51%	39	3.43%	41	20.60%	75^c^	5.91%
Diabetes	15	23.81%	19	8.44%	19	9.84%	22	1.93%	32	16.08%	41	3.23%
Asthma	13	20.63%	17	7.56%	12	6.22%	12	1.05%	22	11.06%	28	2.20%
Eczema	17	26.98%	20	8.89%	9	4.66%	10	0.88%	23	11.56%	29	2.28%
Fibroids	20	31.75%	26	11.56%	12	6.22%	13	1.14%	26^b^	13.07%	36^d^	2.83%

^a ^Trypanosomes includes Chagas disease, leishmaniasis, and African sleeping sickness.

^b ^The combined plant families of Peru and Mali used to treat uterine fibroids and parasitic diseases had a significant difference with p = 0.01911

^c ^The combined plant genera of Peru and Mali used to treat autoimmune and parasitic diseases had a significant difference with p = 0.03987

^d ^The combined plant genera of Peru and Mali used to treat uterine fibroids and parasitic diseases had a significant difference with p = 0.04909

Unfortunately I cannot include further analysis such as family or genera distributions as there are several families with only one or a few species present in both Peru and Mali, so merely naming the family would reveal the species used for a particular disease and break my confidentiality agreement not to publish previously unpublished species uses.

This confirmation of the hypothesis at the high level but with inconsistencies at the lower levels shows that we need to move away from analyzing plant and disease data in these somewhat artificial groupings as they will give us unverifiable results depending on what level the data is analyzed (e.g. species, genus, or family; individual disease or disease category) and which published groupings we used (e.g. the old Malvaceae *sensu strictu *or the new Malvaceae *sensu latu *which includes the old Malvaceae, Sterculiaceae, Tiliaceae, and Bombacaceae [75]). Instead we need to put into practice a system that uses more universal notions of groupings that are not quite so objective and rapidly changing. Using phylogenies to measure evolutionary distance, phytochemistry to gauge how similar disease-treating mechanisms and the compounds in different plants are, and cultural genomic phylogenies can give us more robust information about the relations of plants, diseases, and cultures that should give us more consistent results. It is on these systems that the following theoretical quantitative cross-cultural synthesis technique called "relational efficacy" is based.

## Mathematical background

The hypothesis is that in a database with *N*_*s *_species, *N*_*d *_diseases, and *N*_*c *_cultures, the potential of a certain species *s*, from one culture *c*, to treat a certain disease *d*, (*P*_*s*,*d*,*c*_) should increase with greater phylogenetic proximity of other plants *s' *used to treat related diseases (*R*_*s*,*s*'_), increase with greater etiological proximity of the disease *d' *treated by related plants (*R*_*d*,*d*'_), and increase with less phylogenetic proximity of cultures *c' *using related plants to treat related diseases (*R*_*c*,*c*'_), but it should not increase solely by increasing the size of the dataset. These relatedness factors, discussed further below, would be 1 for two plants, diseases, or cultures that are exactly the same, and would decrease towards 0 as they became less related, e.g., 1/time to their most recent branch point on a phylogenetic tree. Thus we assume that the less related or connected two cultures are, the more likely their discovery of related plants to treat related diseases is an independent event and therefore should increase the plants' medical potential.

The basic formula for the potential *P*_*s*,*d*,*c *_of species *s *to treat disease *d *in culture *c *proposed to meet these conditions is:

$$P_{s,d,c}=\frac{1}{N_{s}N_{d}N_{c}}\sum_{s^{\prime },d^{\prime },c^{\prime }}\frac{R_{s,s^{\prime }}R_{d,d^{\prime }}}{R_{c,c^{\prime }}}$$

where the relatedness factors are summed over all species, diseases, and cultures where species *s *is used to treat disease *d *in culture *c *and species *s' *is used to treat disease *d' *in culture *c'*. *N*_*s *_is the number of species, *N*_*d *_is the number of diseases, and *N*_*c *_is the number of cultures. If a species is not used to treat a disease it does not add to the potential, nor however does it subtract, as it is difficult to make the negative assertion that a particular plant is never used to treat a disease. More interviews may uncover that use. The number of species *N*_*s*_, diseases *N*_*d*_, and cultures *N*_*c*_are divided out to normalize the equation and ensure that the potential of a plant does not increase solely by increasing the sample size. The plant species and disease relatedness values are in the numerator so that the plant's potential increases with *higher *plant and disease relatedness, and the culture relatedness value is in the denominator so that the plant's potential increases with *lower *culture relatedness. It must be emphasized that this is merely an ad hoc formula proposed to meet the assumptions of the hypothesis, but the actual equation will have to be modified with weighting factors, power factors, and/or constants added to it to model the actual data as closely as possible.

The potential could then be summed across all cultures to find the universal potential *P*_*s*,*d *_of a species *s *to treat disease *d*:

$$P_{s,d}=\frac{1}{N_{c}}\sum_{c}P_{s,d,c}$$

where *N*_*c *_is the number of cultures involved. These potentials could be summed over all diseases to determine the universal potential of species *s*:

$$P_{s}=\frac{1}{N_{d}}\sum_{d}P_{s,d}$$

where *N*_*d *_is the number of diseases Further reductions of this potential are possible:

$$P_{c,d}=\frac{1}{N_{s}}\sum_{s}P_{s,d,c},$$

$$P_{d}=\frac{1}{N_{s}}\sum_{s}\frac{1}{N_{c}}\sum_{c}P_{s,d,c},$$

and

$$P=\frac{1}{N_{s}}\sum_{s}\frac{1}{N_{d}}\sum_{d}\frac{1}{N_{c}}\sum_{c}P_{s,d,c}$$

where *N*_*s *_is the number of species, *P*_*c*,*d *_is the potential of culture *c *to treat disease *d*, *P*_*d *_is the potential of disease d to be cured by any herbal remedy in the dataset, and *P *is the overall potential of an entire study. This study potential *P *is a possible way to compare different studies overall success.

Potentials could be summed over all the species in a family or other taxa to determine the values of family, *P*_*f*_, of course normalized to the number of species in the family, or using other techniques such as residuals (Moerman, 1991):

$$P_{f,d}=\frac{1}{N_{s_{2}}}\sum_{s}^{family}P_{s,d}$$

where *N*_*s*,*f *_is the number of species in the family. This should correspond well to previous studies' pinpointing of highly useful or effective families for medicinal plants.

Informant consensus techniques could be used within each culture studied to determine individual cultural reliability weights *w*_*s,c *_that can then be used when summing potentials across cultures or for each plant use:

$$P_{s,d,c}=\frac{1}{N_{s}N_{d}N_{c}}\sum_{s^{\prime },d^{\prime },c^{\prime }}w_{s^{\prime },c^{\prime }}\frac{R_{s,s^{\prime }}R_{d,d^{\prime }}}{R_{c,c^{\prime }}}$$

Alternatively, if informant consensus values are not available for a particular species, disease, or culture because only one or a few healers were interviewed, the normalized length of time the plant remedy has been used by the healers can act as a stand-in to represent how well tested the remedy might be:

$$P_{s,d,c}=\frac{1}{N_{s}N_{d}N_{c}}\sum_{s^{\prime },d^{\prime },c^{\prime }}\frac{t_{s^{\prime },d^{\prime },c^{\prime }}}{t_{\max}}\frac{R_{s,s^{\prime }}R_{d,d^{\prime }}}{R_{c,c^{\prime }}}$$

Where *t*_*s*',*d*',*c*' _is the length of time that species *s' *has been used to treat disease *d' *in culture *c' *in a particular time unit (most likely years), while *t*_*max *_is the maximum amount of time in the same units that any plant has been used in the entire dataset. This would ensure internal consistency within the cultures by giving a higher weight to plants that have been used longer and improve the accuracy of the data, by raising the probability of experimentation and validation within a culture for a particular plant use. If available, informant consensus values would be more accurate since the length of time used is self-reported and therefore more prone to errors as a weighting measure. In my own interviews, I asked each healer how long they in particular had used each remedy and how long they remembered it being used by people in their village, as a backup in case the total number of healers interviewed was too low to use informant consensus on any one remedy. In cases where only a few healers recognized a disease, the informant consensus would most likely not be valid, and the length-of-time-used measure would be used instead for weighting.

**Table 24:** An example of uses of plant species A, B, and C to treat diseases X and Y by culture M, with a '+' if there is a record of a plant being used to treat that disease in that culture and a '-' if there is no such record.

Culture M			
Plants	A	B	C

Diseases			
X	+	+	-
Y	-	+	+

**Table 25:** An example of uses of plant species A, B, and C to treat diseases X and Y by culture N, with a '+' if there is a record of a plant being used to treat that disease in that culture and a '-' if there is no such record.

Culture N			
Plants	A	B	C

Diseases			
X	+	-	-
Y	+	+	-

An example is in order here to demonstrate how these formulae work. Take diseases *X *and *Y*, and the plant species *A*, *B*, and *C *used to treat them in cultures *M *and *N*, as illustrated in Tables 24 and 25. If the relatedness between plants is defined as *R*_*AB *_= 0.5, *R*_*AC *_= 0.7, and *R*_*BC *_= 0.5; the relatedness between cultures is *R*_*MN *_= 0.75; and the relatedness between diseases *R*_*XY *_= 0.3 then

$$\begin{array}{c} P_{A,X,M}=\frac{1}{N_{s}N_{d}N_{c}}\sum_{c^{\prime }=M}^{N}\left(\sum_{d^{\prime }=X}^{Y}\left(\sum_{s^{\prime }=A}^{C}\frac{R_{A,s^{\prime }}R_{X,d^{\prime }}}{R_{M,c^{\prime }}}\right)\right)=\\ \frac{1}{N_{s}N_{d},N_{c}}(\frac{1}{R_{M,M}}\left(R_{X,X}(R_{A,A}+R_{A,B})+R_{X,Y}(R_{A,B}+R_{A,C})\right)\\ +\frac{1}{R_{M,N}}\left(\left(R_{X,X}(R_{A,A})+R_{X,Y}(R_{A,A}+R_{A,B}\right)\right))=\\ \frac{1}{3\cdot 2\cdot 2}(\frac{1}{1}\left(1(1+0.5)+0.3(0.5+0.7)\right)\\ +\frac{1}{0.75}\left(1(1)+0.3(1+0.5)\right))=5.06 \end{array}$$

and so on through the table, yielding Tables 26 and 27 for the calculation of *P*_*s*,*d*,*c*_. When these two tables are summed and normalized for the number of cultures (2), this yields Table 28 for *P*_*s*,*d*_. From Table 28 we can see that species *A *has the highest potential (0.322) to treat disease *X*, and species *B *has the highest potential (0.350) to treat disease *Y*, while species *A *has the highest overall potential (0.322) and would probably be the first species we would want to analyze in the laboratory of the three.

**Table 26:** The calculation of the disease-treating potential *P*_*s*,*d*,*c *_for the hypothetical example of plant species A, B, and C being used to treat diseases X and Y in culture M.

Culture M			
Plants	A	B	C

Diseases			
X	0.316	0.268	0.230
Y	0.313	0.346	0.312
Plant total	0.629	0.614	0.542
Normalized (1/N_D _= 0.5)	0.314	0.307	0.271

**Table 27:** The calculation of the disease-treating potential *P*_*s*,*d*,*c *_for the hypothetical example of plant species A, B, and C being used to treat diseases X and Y in culture N.

Culture N			
Plants	A	B	C

Diseases			
X	0.328	0.271	0.272
Y	0.333	0.354	0.324
Plant total	0.661	0.625	0.596
Normalized (1/N_D _= 0.5)	0.330	0.313	0.298

**Table 28:** The calculation of *P*_*s*,*d *_found by summing and normalizing the calculations of *P*_*s*,*d*,*c *_of culture M and culture N using plant species A, B, and C to treat diseases X and Y.

Both Cultures			
Plants	A	B	C

Diseases			
X	0.322	0.269	0.251
Y	0.323	0.350	0.318
Plant total	0.645	0.619	0.569
Normalized (1/N_D _= 0.5)	0.322	0.310	0.284

If disease *Y *had not been studied in this example, the potentials would have come out as *P*_*A *_= 0.291, *P*_*B *_= 0.240, and *P*_*C *_= 0.240, which we can see is not much different from the normalized row (divided by the number of diseases) in the above table. The potentials have the same rankings of the species and the same magnitude. This shows how the potential is not affected by the size of the dataset (the total number of diseases studied, in this case). Species *A *still has the highest potential of the three.

### Synergy

Mixtures of plants are found in many herbal medicines [65] and the necessary synergy involved in plant mixtures is apparent, among many examples, in the Amazonian hallucinogenic drink *ayahuasca*, usually a mixture of the *Banisteriopsis caapi *(Spruce ex Griseb.) C.V. Morton [Malpighiaceae] containing the monoamine oxidase inhibitors (MAOI) harmine and harmaline, and *Psychotria viridis *Ruiz & Pav. [Rubiaceae], containing the endogenous neurotransmitter dimethyl tryptamine (DMT), neither of which plant would have much effect ingested on their own as the DMT gets broken down in the digestive tract by monoamine oxidase (MAO). However, the combination of the MAOI's in the *B. caapi *blocking the breakdown effect of the MAO, allowing the DMT to enter the brain, creates one of the most powerful natural hallucinogens known. In another example, Lewis et al. [71] found that the combination of two anti-malarial compounds from a Peruvian plant used by the Aguaruna had a 25–33% higher malarial-inhibition effect than the sum of the inhibitions of the individual compounds, i.e., over a quarter of the activity of this compound mixture was synergistic. 5'-methoxyhydnocarpin, found in several species of *Berberis *[Berberidaceae], stopped multi-drug resistant pumps found in *Staphylococcus aureus *from pumping the antimicrobial berberine alkaloids, also found in these same *Berberis *species, out of the cell, the two compounds in combination being much more effective against the microbe as either compound on its own [79]. Raskin and Ripoll [80] give a good review of the many antifungal, antimicrobial, and other synergistic plant compound activities currently known and say there is a great need for such synergistic plant medicines, for instance for multi-drug resistant pathogens and AIDS. Even the United States Food and Drug Administration is accepting clinical trials of botanical drugs with multiple plant components in their Guidance for Industry Botanical Drug Products [81], a change from their former oppositional stance towards botanicals and the difficulty of getting multiple component drugs approved. With all these clear cases of powerful synergistic medicinal effects in plants, how can we deal with the confusing non-linearity of multi-compound and even multiple-plant mixtures with hundreds of potentially active compounds?

**Figure 3:**
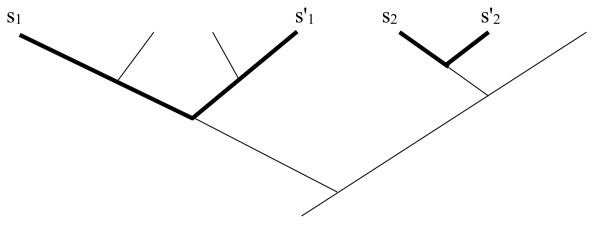
**Plant mixtures from the same clades**. Species used in medicinal plant mixtures may come from similar phylogenetic clades, reinforcing the idea that they are adding similar synergistic phytochemicals to the mixture. Species *s*_1 _and *s*_2 _are from mixture A, and *s*_1_' and *s*_2_' are from mixture B.

The plant potential equations above can be adapted to highlight cases where plants are used synergistically, where plants from one phylogenetic clade are often present in a mixture along with plants from another clade, showing the former plants to be important admixtures even if they never appear alone as a medicine. This would imply that the compounds common in one clade are working together with compounds common in the second clade, one either reinforcing the other, or subduing toxic side effects (see Figure 3).

For comparing a mixtures of two plants, *s*_1 _and *s*_2_, with another mixture of two plants *s'*_1 _and *s'*_2 _the equation would be

$$P_{(s_{1}s_{2}),d,c}=\frac{1}{N_{{s^{\prime }}_{1}{s^{\prime }}_{2}}N_{\text{d}}N_{\text{c}}}\sum_{({s^{\prime }}_{1}{s^{\prime }}_{2}),d^{\prime },c^{\prime }}\frac{{\text{R}}_{s_{1}{s^{\prime }}_{1}}{\text{R}}_{s_{1}{s^{\prime }}_{2}}{\text{R}}_{s_{1}{s^{\prime }}_{2}}{\text{R}}_{s_{2}{s^{\prime }}_{2}}{\text{R}}_{d,d^{\prime }}}{R_{c,c^{\prime }}}$$

with *R*_*d*,*d*' _and *R*_*c*,*c*' _being as above, $R_{s_{x^{\prime }}{s^{\prime }}_{y}}$ being the relatedness of species *s*_*x *_and *s'*_*y*_, and *P*_(*s*1*s*2),*d*,*c *_being the potential of plant mixture *s*_1 _and *s*_2 _for disease *d *in culture *c*. This process can be extended to mixtures of *n *plants with the equation

$$P_{(s_{1}s_{2}\cdots s_{n}),d,c}=\frac{1}{N_{{s^{\prime }}_{1}{s^{\prime }}_{2}\cdots {s^{\prime }}_{\text{n}}}N_{{\text{d}}^{\prime }}N_{{\text{c}}^{\prime }}}\sum_{({s^{\prime }}_{1}{s^{\prime }}_{2}\cdots {s^{\prime }}_{n}),d^{\prime },c^{\prime }}\frac{\prod_{x=1,y=1}^{{\text{N}}_{\text{s}}{\text{,N}}_{{\text{s}}^{\prime }}}R_{s_{x}{s^{\prime }}_{y}}R_{d,d^{\prime }}}{R_{c,c^{\prime }}}$$

with the combinatorics of plant-plant relatedness products increasing rapidly with the number of the plants in the mixture. Yet this should still be a tractable way to pinpoint those plants that are aiding the action of another plant in a medicinal mixture, i.e., those plants that appear in high-potential mixtures, but are not recorded as being used alone for the same disease.

### Uncertainty

Even if all the medicinal plants collected cannot be identified to species with confidence, something that is quite common in ethnobotany where collaborators may just give the researcher the ground-up leaves or roots of a plant to identify [65], these data can still be used by employing resampling statistics methods. Most ethnobotanists eliminate data on plants they cannot fully identify, but if a plant is identified as a particular genus, or from the plant's common name it can be inferred that it is one of several possible unrelated species, this information can be used to derive medicinal efficacy potentials for the plant. Common-name uncertainty is much more difficult to use than uncertainty of several species within one genus, as the actual species corresponding to a common name could be in any of several disparate genera or families, or just completely misidentified by the collaborator. The potential value calculated will not be as exact as if there is a species-level identification for the plant. Instead, it will have a range of values or confidence intervals derived using resampling statistics techniques, where the potential efficacy of each collected plant is calculated thousands of times while resampling from collected data to give potential values for the different combinations of possible plant species identifications. These thousands of calculated potentials are then used to find an average potential and an error range for those unknown plants. In the case of common-name uncertainty, if the dataset is small and the uncertain species are key to the potential calculations (i.e. when one of the possible species is used to treat a disease closely related to other diseases and is closely related to many other species in the dataset), the calculated potentials may be in several discrete ranges rather than one as the input species' relatedness values would be quite disparate. The more incompletely identified plants there are in a dataset, the more uncertainty there is (e.g. from many possible species corresponding to a common name), and the greater the error ranges of the plant potential will be, but this will still often be enough to rank it in a list of plants with the highest potentials. This usage of incomplete data in ethnobotany would be quite useful in many studies.

### Non-native species

Medicinal plant uses that have been introduced into a culture from another culture must be eliminated from the data, as they are less likely to be *independent *discoveries of the same plant use. We do not want cases such as jambol (*Syzygium cumini *(L.) Skeels [Myrtaceae]) being used in European herbal medicine to treat diabetes, as this is a native of Asia and therefore a case of transmitted information and not independent discovery. Because of this, a good understanding is required of the introduced vs. traditional plant uses and how this medicinal knowledge is disseminated. Introduced plant uses can be determined by comparing the plant use data gathered to any previous ethnobotanical surveys done in the cultures being studied to see if their uses are changing over time. If the collaborators in the studied cultures describe these new uses as being from outside their culture, as Campos et al. [32] has documented with the Yawanawá and Kaxinawá in Brazil, these should be considered introduced uses, rather than self-discovered uses, and eliminated from the data. If no previous ethnobotanical surveys exist for these cultures, floras of the area which describe whether plants are native or introduced can be used to eliminate any uses of introduced plants from the data. Newly-discovered medicinal uses of *introduced species*, as opposed to *introduced uses *of plants, might be worth keeping in the database if the two subtle cases can be differentiated, as they may be tested uses and just as valid as uses of native species. An introduced species being used in its introduced area A for a disease X related to the disease Y for which it is used in the species' native area B is even more ambiguous, as one must definitively determine that the culture of area A does not consider diseases X and Y to be related and therefore that this is an introduced use. Bennett and Prance [28] discuss introduced medicinal species at length, saying they are well represented in the pharmacopoeia of Northern South America. This is not to claim that introduced species are ineffective, but rather that it is difficult to say how long they have existed in a certain area and therefore whether they have been truly tested there and if this use is an independent discovery from the uses in the plants' native area. Clues such as local plant names and introduced species' ranges cannot clearly date the species' introduction and therefore the amount of experimentation with the plant.

The local plant names can be some indication of an introduced use, i.e., local plant names not in the local language provides some evidence, albeit not definitive, that the use has traveled along with the name from the plant's native area to the introduced area [82,29]. Introduced uses moving from one native area of a plant to another native area of the plant where there are local names in both areas are more complicated, as the use may transfer without the name. Therefore, it is important to look at the natural range and local names of the plants considered.

As an example, in Peru the Asháninka use the Indian-native neem tree (*Azadirachta indica *A. Juss. [Meliaceae]) to treat diabetes (personal observation, 2003), a use that is found in India as well. When asked how this use came about, Raúl Casanto Shingari, the chief of the Asháninka village of Paititi said that the neem tree was introduced to certain areas of Peru, such as the Amazon city of Pucallpa, when some Peruvians went to Costa Rica for an agriculture workshop, where they learned of the excellent pesticidal qualities of neem. Not having heard anything about its diabetes-treating qualities, one of the Asháninka who had diabetes tried a tea of it because of the bitter taste of the neem leaves and saw a rapid improvement in his condition. He then told his companions about this use, and its use spread around the community. If all of the facts of Raúl's story are correct, this would be a case of independently discovered use of an introduced plant, and can be included in the dataset. In Mali, neem is very common in large towns as a street tree that was introduced in 1950, the year of Malian independence, and is therefore called "mali yirini" or "plant of Mali", yet none of the 15 healers interviewed in Mali who were very familiar with the plant used neem to treat diabetes. In this case, even if neem had been used for diabetes or other diseases, because of the wide distribution of neem and much higher immigration to the area from the Indian subcontinent than to Peru, it would have been very difficult to determine if this was an introduced use, or an independently-discovered use of an introduced plant.

## Plant relationships

The idea that certain taxonomically related plant groups (taxa) have a higher occurrence of medicinally active compounds in them we will call "taxon predominance" here. The measurement of taxon predominance is somewhat problematic because taxonomic rank is a somewhat arbitrary objective construct that leads to anomalies: when simply counting medicinal plants in certain families, it emphasizes larger families, and when finding percentages of medicinal plants in families, it emphasizes small families [4]. Although these anomalies can be resolved somewhat by looking at residuals in predicted percentages, as Moerman has, there are still a few problems. First, considering if two plants share a taxon is a binary question- are plants *A *and *B *in the same family or not? – when we would really like a continuous measure that would give us more information from which to derive descriptive statistics – how closely related are plants *A *and *B*? Second, these residuals are not normally distributed, making it difficult but not impossible to analyze the significance of the differences in residuals as they violate the assumptions of standard statistical analyses [74,83]. A randomization technique such as Monte Carlo simulation or resampling statistics may get around this issue, and this has been done on a large database of Native American medicinal plants, finding that plant families with statistically significantly low or high number of medicinal species do not correlate with the families' species number and that most likely certain families evolved chemical adaptations suited to their ecological setting that lead them to be selected more often by the Native Americans as medicinal plants [84].

However, despite the statistical rigor of this last approach, the third problem is that looking at taxon predominance deals with taxa, which do not circumscribe consistent degrees of proximity at the same ranks (taxa levels), i.e., two plants that are in family *A *are not necessarily as closely related as two plants in family *B*. If a taxonomist prefers to split large plant families, two species in different families may in fact be more closely related than two species in the same genus in another large family, as Avise and Johns [85] have discussed with the relatively recently evolved, small group of primates (7 species) being split across several families compared with the speciose, earlier-branching fruit flies with 12 species being grouped into one genus. Avise and Johns have attempted to address this somewhat by proposing a system in which different taxonomic ranks would strictly represent a measure of evolutionary relations by indicating the time since divergence from the taxa's common ancestor. For instance, genera would be species groups that branched off from each other 5 million years ago, families would have a branch point of 20 million years ago, and orders 45 million years ago. This proposed system, however, does not take into account different rates of evolution in different branches under different evolutionary pressures.

To implement this we would have to know the time of the evolutionary branch points for all described plants to be able to put them in the correct taxa, but this information is available for only a small percentage of known plants. One technique that has had some success in putting dates on evolutionary events uses molecular clocks to date divergence points, where the rate of DNA or RNA mutation since a divergence point is measured and calibrated against a known standard mutation rate [86,87]. This seems to work best only in small plant groups, however, as the supposed constant rate of DNA mutation across large groups seems to break down, considering the fact that plants evolve at different rates at different times in history under different environmental conditions [88,89]. Inventive fixes to this variation can be performed, such as using plant fossil data and the proposed taxonomy phylogenies of the flowering plants [90] to push back the divergence date of sister plant taxa that are known to share a common branch point [91]. The "Deep Time" project [92,93], an attempt to bring together data from molecular and morphological systematics, paleobotany, and geology to date many of the angiosperm evolutionary divergence events, will collect much of the data needed in one place for finding plant relationships by their date of divergence from their most recent common ancestor. In the near term, the inaccuracies of this system would allow it to only be used for higher ranks such as families or orders, so for lower ranks another system is needed.

A simple technique for measuring the genes common to two plants, like *re-annealing *[94] where the time for separated DNA strands from the two plants to reconnect or "re-anneal" with each other is determined, could be performed for plants in this study. This is a fast procedure, and the re-annealing times derived from this could be used as a measure of plant relatedness, but the number of experiments necessary would quickly skyrocket as the combinatorics of comparing each plant to all the others in the study increases with large sample sizes. Comparing *n *plants would require *n!/(n-2)!2 *physical experiments in the test tube, reannealing each species' DNA with every other species' DNA, which for even 50 plants would mean 1225 physical comparison experiments. Creating quick chemical fingerprints or metabolite profiles [95] of all the plants in the study can be determined using high-performance liquid chromatography (HPLC) [96,97], which monitors the diffusion time and spectra of compounds in a plant extract as they diffuse in a solvent through a column filled with different substrates, or diffusion-ordered (DOSY) nuclear magnetic resonance (NMR) imaging [98,99] which uses NMR to create a fingerprint of multi-compound plant extracts by mapping how these compounds diffuse over time under magnetic excitation. Both these techniques would be a relatively easy way to avoid the proliferating combinations of plant comparisons in re-annealing as the fingerprints for each plant are recorded on a computer, where calculating the relationships among all the combinations of plant fingerprints could be done in minimal time. For 50 plants, only 50 physical readings need to be done with HPLC or DOSY NMR, and the 1225 comparisons are calculated in the computer using the output of these 50 tests, i.e., the data can be reused. With re-annealing, every comparison of two plants must be done physically in the test tube, whereas with chemical fingerprinting the comparisons are done on the computer.

This chemical-fingerprint approach to determining the relationships of plants has two advantages over a phylogenetic molecular-clock approach. First, a chemical comparison gets more directly at what we are looking for in the plants - are two plants sharing some secondary metabolites that would act in a similar way in the human body to treat a disease - rather than using the proxy for metabolite similarity of genetic similarity that phylogenies represent. There are many steps (promotion, transcription, deletion, folding, and synthesis pathways, to name a few) that separate similar DNA from similar metabolites. Second, for plants for which there is no existing description of relationships - chemical, phylogenetic, or otherwise - it is much easier to derive metabolite similarity of a random sampling of plants from across the plant kingdom through fingerprints, than to derive a dated phylogeny for these isolated plants without the context of their genera, families, and orders. The fingerprint approach has the disadvantage that one collection of a plant may only represent the metabolite fingerprint for that time of day, season, location, stress level or plant part, as metabolites can vary widely in the same species with all these dependent variables [96]. This problem can be worked around, however, by sampling the plant part, time, and location as the collaborators do, since this represents the metabolites they are using in their herbal medicines, or by taking a cross-section of all the parts, times of day, seasons, and locations available to the healers and grouping all these samples of one species as one plant when doing the fingerprint, as a way to try to get all the possible metabolites that might be present in this species over all conditions. The latter scheme may be impractical due to the immense amount of collection time necessary, however.

Metabolite fingerprinting will work best with closely related plants species, as plants in different families or orders can often have such different secondary compounds that the extraction methods must be quite different and fingerprint data will have little or no similarities. However, the fingerprint method of determining plant relatedness will complement the dated phylogeny method, as dated phylogenies have been determined mainly for the broader scale of orders and families, but not between genera and species as of yet. Therefore, the dated phylogeny method of relatedness should be used to determine broader scale relationships for those families for which it exists, and, if needed, the fingerprint method can be used to fill in at the smaller scale. A calculation of both phylogenetic and metabolite relatedness for the same set of species can be used to calibrate these different systems to each other, if there is some area of overlap.

Another assumption of this technique is that phytochemicals are conserved across genera, families, or orders; there is a basis for this, as phytochemicals have been used in the past as a trait to create phylogenies in the field of plant chemosystematics [100]. Many compounds are found across entire families or orders, such as cyclopentenoid cyanogenic glycosides found in the Achariaceae, Passifloraceae, Turneraceae, and Malesherbiaceae within the order Malpighiales; betalains in the Caryophyllales; and the sesquiterpene lactones common in the Asteraceae [101]. Some compounds are found only in certain genera, as with hypericin in *Hypericum sp*. [Clusiaceae] [102] and betulin in the *Betula *genus [Betulaceae] [101]. This most likely explains people's tendency to concentrate their medicinal plants in certain families [2]; they are realizing that some effects [103] or tastes [104] of a group of plants are similar and therefore they are using other members of that plant group to treat their diseases as they most likely contain similar disease-treating components. Balunas [105] has done an extensive analysis of how the percentage of active plants and average 50% effective concentration (EC_50_) values of anticancer activity in large plant collections from around the world vary with the plant part, collection location, and plant family, showing the interesting trends that percent of active plants is not higher in areas with higher biodiversity, but is higher in the Clusiaceae, Elaeocarpaceae, Meliaceae, and Rubiaceae than other families and higher in roots and below-ground collections than above-ground collections.

## Disease relationships

Little to no research has been done that considers the issue of treating related diseases with related plants. Some diseases in past studies may be connected such as different types of infectious diseases like wound infections and thrush, which may actually be caused by different taxa of bacteria. If we look more deeply into the Western classification and causes of diseases [106], we realize that seemingly unrelated diseases may have the same underlying cause and be treated in similar ways. For instance, it would appear that eczema, diabetes, and asthma are very different diseases, but they are all in fact auto-immune syndromes – the body turning against and attacking itself, in one case in the skin, another in the pancreas, and the third in the respiratory system [107]. Once again, for the proposed approach we need to be able to measure the relatedness of the diseases, regardless of whether they are due to genetic, infectious, or environmental causes.

The relatedness of two diseases is perhaps the hardest of the three relatedness measures to delineate, as diseases did not all evolve from a common ancestor and therefore are not linked by a phylogeny as cultures and plants are. We can say that two different bacterial infections are closely related, but how can we say how closely a bacterial infection and sickle cell anemia are related? One is caused by an invading organism and the other by genetics.

Exacerbating this problem is the fact that Western doctors classify diseases mainly by the body system affected, such as cardiovascular, brain, or bone diseases, because doctors use the symptoms within these body systems to diagnoses diseases [106]. Some diseases are grouped together by their causes, such as autoimmune diseases, but as diseases are treated more often for their symptoms than for their underlying causes, this is usually not the case. One approach to linking diseases with their different base causes is to look for patterns in the existing medicinal plants that are laboratory-proven to effectively treat different diseases in order to reveal the related mechanisms of causation and treatment of diseases, avoiding plants that are part of the main database being analyzed so as not to be tautological. Specht [108,109] has done this type of analysis using cladistic computer programs using the parsimony algorithm to determine how plant families are related by the diseases they are used to treat (a method that could be termed pathotaxonomy, analogous to chemotaxonomy), and how diseases are related by the plant families used to treat them (which we will call "plant-based disease taxonomy" or PBDT).

There is some evidence of similar diseases being treated with closely related plants, as Lukhoda, Simmonds et al. [110] have shown by looking at the problem pathotaxonomically that similar disease-treating characters group together on a phylogeny of *Plectranthus *species [Lamiaceae]. Senchina et al. [111] performed a similar phenetic analysis of several *Echinacea *species [Asteraceae], showing that some of the immunomodulatory characteristics of the species align with one interpretation of *Echinacea*'s phylogenetic clades if both immune boosting and immune suppressing characteristics are taken into account, showing that we must look at not just one but rather many medicinal actions to see a correlation with the plant phylogenies, and to be able to use the plant phylogenies as an indication of a shared disease treatment mechanism or vice versa. Daly and Stevenson [112] have extended the PBDT method to grouping diseases by the plant species used to treat them. In this case, if a subset of the same plants is used to treat the same diseases, the diseases are more likely to be caused by a similar metabolic system in the human body that is being affected therapeutically in similar ways by these similar plants. This technique uses algorithms borrowed from plant taxonomy to find patterns of related diseases where diseases are treated by the same plant. For instance, in Guatemala, the Neotropical herb *tres puntas *(*Neurolaena lobata *(L.) Cass. [Asteraceae]) is used there (personal observation, 2000) and shown in the laboratory to treat malaria, diabetes, and dengue fever [113-116]. Therefore, under the shared-plant-treatments technique, these three diseases would be considered to have some relation, depending on other plants also used to treat these diseases.

In order to avoid circular reasoning while using the PBDT method to find disease relatedness values, the plants in the main study dataset should not be used to find disease relations, but rather plants that have already been studied in the laboratory and have been shown to be effective against the diseases being studied. The NAPRALERT (Pharmacological Sciences (PCRPS), College of Pharmacy, University of Illinois) and MEDLINE [117] databases are good sources on laboratory and clinically tested medicinal plants for this technique. Using laboratory-proven medicinal plants to find these patterns could permit the creation of a broader disease taxonomy and estimate disease relatedness. If there is not a sufficient number of laboratory-tested plants to link the diseases being studied, the diseases analyzed would have to be limited to comparisons of groups of phylogenetically related diseases, such as among the infectious Protista kingdom parasite diseases (malaria, Chagas' disease, African sleeping sickness, and leishmaniasis). In fact, one good test of the PBDT technique is to compare the relationships it determines for the set of infectious Protista diseases to the phylogenetic relationships determined by systematists for the Protista species. If this comparison of the PBDT and phylogenetic methods of determining disease relatedness validates the PBDT method, it can be used to tie together all the diseases, otherwise the analysis should be limited to within the easily related disease classes.

A third option for determining disease relations is the relatively new genetic drug-disease connectivity map [118] derived from the human genome project data that show how diseases and pharmaceuticals that are used to treat them affect similar genes. This would only work for the diseases with a genetic basis or predisposition (uterine fibroids, eczema, asthma, and diabetes in this study) and a measure of relatedness would have to be derived from the connectivity network. The relatedness values from this system can again be compared to the relatedness values derived from the PBDT as a way to validate and calibrate this method.

## Cultural relationships

To determine cultural relatedness, it has been suggested to simply look at geographic distance between the two cultures, but this is problematic as geographic barriers such as mountains and oceans that slow the transmission of cultural knowledge are hard to factor in. Are the indigenous groups of southern Argentina and Chile really as similar to those of South Africa, at 7,100 km distance, as they are to the people of Costa Rica, also 7,100 km away? These cultural barriers are not very easy to quantify.

Alternatively, evolutionary language trees could be considered, as they are a fairly complete record of the intermingling of different cultures and passing of information such as herbal remedies. Glottochronology is a technique that can be used to date language phylogenies using common words between languages, called *cognates *[119], but glottochronology is not considered valid past 5,000 years ago for native North American languages [120] and not past 6,000 years ago for Indo-European languages [121], including English and Hindi. This means that glottochronology would not work for the distant cultures of Peru and Mali being considered.

Cultural phylogenies have been developed based on multiple genetic comparisons that are probably valid past earlier dates. These genetic phylogenies match quite closely with language phylogenies and actually may be a better indicator of cultural knowledge transmission than language phylogenies as languages can hybridize quite rapidly, e.g., creoles and pidgins [119]. Given that these genetic cultural phylogenies are dated, they will be used to calculate cultural relatedness by using a metric such as 1/time to the most recent common ancestor of the two cultures. This genetic cultural-relatedness method appears to be much more viable for the distantly related cultures under study than glottochronology, and is currently being updated with National Geographic's Genographic project [122,123] which should cover the Asháninka and Malinké groups that are part of this study that Cavalli-Sforza has not.

## Discussion

In the end, the best data on relationships will come from a combination of metabolite fingerprinting and dated phylogenies like "Deep Time" for plants; disease descriptions, relations, and shared-plant treatments for diseases; and genetic phylogenies for cultures. Advances in these techniques will likely come up that can be integrated as well as the following extensions of relations of plant parts used, using existing databases, model validation and prediction. It should be pointed out that some measures of relatedness will be more accurate than others, and only some of these potential measures, out of a realm of many possibilities, are described here. However, the equations that synthesize these relations into measures of potential medicinal efficacy of each plant should function regardless of how the relations are measured.

It may be possible to add additional factors for the relatedness of the plant part used (root, bark, wood, leaves, flowers, fruit, seeds, or combinations thereof), extraction method (decoction; alcohol, water, or oil tincture; infusion; entire plant), season harvested, companion plants, and growth habit, to the above equations in order to refine their accuracy if a suitable measure of these relations could be determined. There is no immediately obvious metric, for instance, of how the different compounds found in the roots vs. the leaves of different species might be related, analogous to the relatively simple metrics of the phylogenetic distances for species, culture, and diseases. Unless many different plant parts from unrelated species can be tested for efficacy to derive some measure of the average relatedness between the compounds in leaves and roots, for instance, it may be difficult to include factors such as this in the calculations.

Any published ethnobotany study or database can be integrated into the data to broaden the coverage and increase the accuracy of the data. For instance, the United States Department of Agriculture's phytochemistry and ethnobotany database [124], the Native American Ethnobotany Database [125], and the culturally more similar International Ethnobotany Database [126] would allow different cultures that have not been studied firsthand to be included in the medicinal potential analysis, although different interview and research methodologies may cause problems in a unified analysis of these databases.

Once data on a sufficient number of medicinal plant species, diverse cultures, and related diseases has been collected, the manner that the three factors of plant, disease, and cultural relatedness interact in the mathematical model can be assessed. The formulas presented above are ad hoc and therefore need to be validated or modified. This can be done by performing a consistent evaluation of each plant species' medical efficacy, via either bioassays or literature searches, and seeing how the efficacy correlates with the relatedness of the plants, diseases, and cultures. Existing studies have tried to make a standard measure of efficacy by grading previous lab or clinical studies on plants from the literature as "not effective", "effective", or "highly effective," but of course, this always introduces the grader's bias [3]. With possible access to one of the large ethnobotanical databases such as those of Duke or Moerman, a quick verification of the system could be performed using literature studies as a sort of verification, but the vast differences in the way plants' medicinal efficacies are tested in the existing literature makes this approach problematic. Instead, a consistent set of efficacy studies on the plants would give more reliable verification of the system. It would also be interesting as another form of validation to see how the index proposed here correlates with other ethnobotanical indices such as informant consensus values and relative importance as has been done with several existing indices [13,15].

Measuring efficacy across diseases can be difficult as, for instance, one cannot reliably compare EC_50 _values from an antimalarial assay to the EC_50 _values for a diabetes assay. General disease-treating efficacy could be measured using bioassays such as the brine shrimp assay for bioactivity, which can be used across different diseases [3], but this is inaccurate, as it only tests for certain types of biological activity that might occur in the human body. Therefore, for validation purposes, bioassay tests should be used for comparison of plant efficacy activity only within one disease, and the efficacy test by literature review such as Trotter and Logan's [3] should be used to compare between different diseases. One way to adjust for differences between diseases is to factor in the efficacy of the dose a healer usually administers for particular disease, or calculate how this efficacy compares with the effective dose of a proven standard pharmaceutical, i.e. how close does the dose of a plant given traditionally come to an effective dose.

Prediction of unexplored but effective medicinal plants will be possible, perhaps for the first time in this field, as the potential of any plant in a dated phylogeny can be calculated, not only those that are actually used in treatments. Plant species with no reported medicinal use can easily be plugged into the quantitative system based on their relations to other plants with known uses, producing a measure of the medicinal potential for the unreported plant that may be within the range of potentials for reported plants. If these plants are in the top of the range of computed potentials, they should be considered for laboratory analysis for true efficacy and they may turn out to be just as effective if not more so than reported plants.

## Conclusion

A preliminary quantitative cross-cultural analysis of Peruvian and Malian medicinal plants has shown that some of the prerequisite hypotheses of shared plant remedies are true, but a more refined analysis is necessary. A new theoretical mathematical methodology of "relational efficacy" has been introduced that ethnobotanical researchers can use to estimate the potential of the plants they have studied before the plants have been fully analyzed in a laboratory. Once this system is validated, it should also allow effective comparison between studies by looking at the difference in the overall potential of all the medicinal plants in each study or the potential of particular species between studies. Thus, this system will be able to synthesize many cultures' medicinal plant knowledge to pinpoint plants with a high potential for being medically effective, save limited laboratory time and resources, and predict species that may have great disease-treating potential that have never before been considered in any culture.

## References

[B1] McClatchey W, Bridges KW (2002). Strong Inference in Ethnobotany. 43rd Annual Meeting of the Society for Economic Botany.

[B2] But PP, Hu S, Kong YC (1980). Vascular plants used in Chinese medicine. Fitoterapia.

[B3] Trotter RT, Logan MH, Etkin NL (1986). Informant consensus: A new approach for identifying potentially effective medicinal plants. Plants in indigenous medicine and diet.

[B4] Moerman DE (1991). The medicinal flora of native North America: An analysis. Journal of ethnopharmacology.

[B5] Lawrence A, Phillips O, Ismodes A, Lopez M, Rose S, Wood D, Farfan A (2005). Local values for harvested forest plants in Madre de Dios, Peru: Towards a more contextualised interpretation of quantitative ethnobotanical data. Biodiversity and Conservation.

[B6] Phillips OL, Gentry AH (1993). The useful woody plants of Tambata, Peru I: Statistical hypotheses tests with a new quantitative technique. Economic Botany.

[B7] Lewis WH, Elvin-Lewis MP (1995). Medicinal Plants as Sources of New Therapeutics. Annals of the Missouri Botanical Garden.

[B8] Balick MJ, Cox PA (1996). Plants, people, culture: the science of ethnobotany.

[B9] Ubillas R (1994). SP-303, an antiviral oligomeric proanthocyanidin from the latex of Croton lechleri (Sangre de Drago). Phytomedicine.

[B10] McClatchey W (2005). Medicinal Bioprospecting and Ethnobotany Research. Ethnobotany Research & Applications.

[B11] Kapur SK, Shahi AK, Sarin YK, Moerman DE (1992). The medicinal flora of Majouri-Kirchi forests (Jammu and Kashmir State), India. Journal of Ethnopharmacology.

[B12] Friedman J, Yaniv Z, Dafni A, Palewitch D (1986). A preliminary classification of the healing potential of medicinal plants, based on a rational analysis of an ethnopharmacological field survey among bedouins in the Negev Desert, Israel. Journal of Ethnophamacology.

[B13] Albuquerque UP, Lucena RFP, Monteiro JM, Florentino ATN, Cecília de Fátima C. B. R. Almeida (2006). Evaluating Two Quantitative Ethnobotanical Techniques. Ethnobotany Research & Applications.

[B14] Andrade-Cetto A, Becerra-Jiménez J, Martínez-Zurita E, Ortega-Larrocea P, Heinrich M (2006). Disease-Consensus Index as a tool of selecting potential hypoglycemic plants in Chikindzonot, Yucatan, México. Journal of Ethnopharmacology.

[B15] Reyes-García V, Vadez V, Tanner S, McDade T, Huanca T, Leonard WR (2006). Evaluating indices of traditional ecological knowledge: a methodological contribution. J Ethnobiol Ethnomedicine.

[B16] Johns T, Kokware JO, Kimanani EK (1990). Herbal remedies of the Luo of Siaya District, Kenya: establishing qualitative criteria for consensus. Economic Botany.

[B17] Begossi A (1996). Use of Ecological Methods in Ethnobotany - Diversity Indexes. Economic Botany.

[B18] Bruni A, Ballero M, Poli F (1997). Quantitative Ethnopharmacological Study of the Campidano Valley and Urzulei District, Sardinia, Italy. Journal of Ethnopharmacology.

[B19] Galeano G (2000). Forest Use at the Pacific Coast of Choco, Colombia - A Quantitative Approach. Economic Botany.

[B20] Johns T, Mahunnah RLA, Sanaya P, Chapman L, Ticktin T (1999). Saponins and phenolic content in plant dietary additives of a traditional subsistence community, the Batemi of Ngorongoro District, Tanzania. Journal of Ethnopharmacology.

[B21] Johns T, Mhoro EB, Sanaya P, Kimanani EK (1994). Herbal Remedies of the Batemi of Ngorongoro District, Tanzania - A Quantitative Appraisal. Economic Botany.

[B22] Johns T, Faubert GM, Kokwaro JO, Mahunnah RLA, Kimanani EK (1995). Anti-giardial activity of gastrointestinal remedies of the Luo of East Africa. Journal of Ethnopharmacology.

[B23] Lewis WH, Elvin-Lewis M, Gnerre MC, W. DF, Hedberg I (1988). Role of Systematics When Studying Medical Ethnobotany of the Tropical Peruvian Jivaro. Systematic Botany—A Key Science for Tropical Research and Documentation.

[B24] Quan X, Young D, Jie K (1991). A computer assisted comparison between traditional chinese medicine and the indigenous medicinal sytems of Tibetan, Nepal, and India: Mexico City, Mexico.. meeting of the International Society for Ethnobiology.

[B25] Mace R, Pagel M (1994). The comparative method in anthropology. Current Anthropology.

[B26] Ostraff M (1995). Dissemination of tapa cloth technology throughout Polynesia using a Fuzzy set alternative to clustering methods: Ithaca, NY..

[B27] Weiss J (1998). Diagnostic concepts and medicinal plant use of the Chatino (Oaxaca, Mexico) with a comparison of Chinese medicine. PhD Dissertation.

[B28] Bennett B, Prance G (2000). Introduced plants in the indigenous pharmacopoeia of Northern South America. Economic Botany.

[B29] Johnson LM (2006). Gitksan medicinal plants-cultural choice and efficacy. Journal of Ethnobiology and Ethnomedicine.

[B30] Palmer C (2004). Plantago spp. and Bidens spp.: A case study of change in Hawaiian herbal medicine. Journal of Ethnobiology.

[B31] Palmer CT (2004). The Inclusion of Recently Introduced Plants in the Hawaiian Ethnopharmacopoeia. Economic Botany.

[B32] Campos MT, Ehringhaus C (2003). Plant virtues are in the eyes of the beholders: a comparison of known palm uses among indigenous and folk communities of Southwestern Amazonia. Economic Botany.

[B33] Cox PA, Cox PA, Banack SA (1991). Polynesian herbal medicine. Islands, plants, and polynesians: an introduction to polynesian ethnobotany.

[B34] Lenaerts M (2006). When Inter-ethnic Botanical Borrowing does not rely on Obvious Efficacy: Some questions from Western Amazonia. Ethnobotany Research & Applications.

[B35] Heinrich M, Ankli A, Frei B, Weimann C, Sticher O (1998). Medicinal plants in Mexico: healers' consensus and cultural importance. Social Science & Medicine.

[B36] Bennett BC (2007). Doctrine of Signatures:  An explanation of medicinal plant discovery or dissemination of Knowledge?. Economic Botany.

[B37] Adjanohoun EJ, Ake Assi L, Floret JJ, Guinko S, Koumarc M, Ahyi AMR, Raynal J (1980). Contribution aux études ethnobotaniques et floristiques au Mali. Médecine traditionnelle et pharmacopée.

[B38] Boudet GG, Lebrun JPJP (1986). Catalogue des plantes vasculaires du Mali. Etudes et synthèses de l'IEMVT, 0297-4444.

[B39] Malgras D (1992). Arbres et arbustes guérisseurs des savanes maliennes. Economie et développement (Paris, France).

[B40] Daly DC, Foster R, León B, Olson D, Dinerstein E, Castro G, Maraví E (1996). Southwestern Amazon moist forest-Southwest, Juruá, Purus-Madeira -- Peru, Brazil, Bolivia. Identifying Gaps in Botanical Information for Biodiversity Conservation in Latin America and the Caribbean.

[B41] Gordon RG (2005). Ethnologue: Languages of the World, Fifteenth edition.

[B42] Narby J (1989). Visions of land: the Ashaninca and resource development in the Pichis Valley in the Peruvian Central Jungle. Anthropology.

[B43] Keplinger K, Laus G, Wurm M, Dierich MP, Teppner H (1999). Uncaria tomentosa (Wild.) DC.— Ethnomedicinal use and new pharmacological, toxicologival and botanical results. Journal of Ethnophamacology.

[B44] Anonymous (1993). Victims in the forest. The Economist.

[B45] Brown MF (1996). On resisting resistance. American Anthropologist.

[B46] Foster D (1990). No road to Tahuanti. Mother Jones.

[B47] Holligan de Diaz-Limaco J (1998). The path to freedom. Geographical.

[B48] Narby J (1993). Smoking out the spirits. Buzzworm.

[B49] Simpson J (1993). To the beginning of the world. World Monitor.

[B50] Brown MF, Fernández E (1991). War of shadows: the struggle for utopia in the Peruvian Amazon.

[B51] Gagnon M, Hoffer W, Hoffer M (1993). Warriors in Eden.

[B52] Veber H (1998). The salt of the Montana: interpreting indigenous activism in the rain forest. Cultural Anthropology.

[B53] Weiss G (1969). The cosmology of the Campa Indians of Eastern Peru. Anthropology.

[B54] Keplinger K (1993). Der Baum, der einem Mann ein Kind schenkte (The tree the one man gave a child).

[B55] Keplinger K (1993). Das Shevátari. Eine vergessene Schrift aus dem peruanischen Urwald (The Shevátari. A forgotten writing from the Peruvian jungle).

[B56] Anderson RJ (2000). Ashéninka stories of change. SIL International Publications in Sociolinguistics.

[B57] Lenaerts M (2006). Substances, relationships and the omnipresence of the body: an overview of Asheninka ethnomedicine (Western Amazonia). J Ethnobiol Ethnomedicine.

[B58] Daly DC, Silveira M, collaborators Primerio Catálogo da Flora do Acre, Brasil/First Catalogue of the Flora of Acre, Brazil..

[B59] Schaffer M, Cooper C (1980). Mandinko: the Ethnography of a West African Holy Land.

[B60] Stauble N (1986). Ethnobotany of Euphorbiaceae of West Africa. Journal of ethnopharmacology.

[B61] Arbonnier M (2002). Arbres, arbustes et lianes des zones sèches d'Afrique de l'Ouest.

[B62] Benson DA, Karsch-Mizrachi I, Lipman DJ, Ostell J, Rapp BA, Wheeler DL (2000). GenBank. Nucleic Acids Res.

[B63] Federhen S, Harrison I, Hotton C, Leipe D, Soussov V, Sternberg R, Turner S The National Center for Biotechnology Information (NCBI) Entrez taxonomy database. http://www.ncbi.nlm.nih.gov/entrez/query.fcgi?db=taxonomy.

[B64] Wheeler DL, Chappey C, Lash AE, Leipe DD, Madden TL, Schuler GD, Tatusova TA, Rapp BA (2000). Database resources of the National Center for Biotechnology Information. Nucleic Acids Res.

[B65] Balick MJ, Kronenberg F, Ososki AL, Reiff M, Fugh-Berman A, O'Connor B, Roble M, Lohr P, Atha D (2000). Medicinal plants used by Latino healers for women's health conditions in New York City. Economic Botany.

[B66] Bletter N (2006). Talking books: A new method of returning ethnobiological research documentation to the non-literate. Economic Botany.

[B67] Gentry AH, Conservation International. (1996). A field guide to the families and genera of woody plants of northwest South America (Colombia, Ecuador, Peru), with supplementary notes on herbaceous taxa.

[B68] Milliken W, Albert B (1996). The Use of Medicinal Plants by the Yanomami Indians of Brazil. Economic Botany.

[B69] Milliken W (1997). Traditional Anti-Malarial Medicine in Roraima, Brazil. Economic Botany.

[B70] Alexiades MN (1999). Ethnobotany of the Ese Eja. PhD Dissertation.

[B71] Lewis WH, Lamas G, Vaisberg A, Corley DG, Sarasara C (1999). Peruvian Medicinal Plant Sources Of New Pharmaceuticals (International Cooperative Biodiversity Group-Peru). Pharmaceutical Biology.

[B72] Laird SA (2002). Biodiversity and traditional knowledge: equitable partnerships in practice. People and plants conservation manuals.

[B73] Lewis ME (2006). Evolving concepts related to acheiving benefit sharing for custodians of traditional knowledge.. Ethnobotany Research and Applications.

[B74] Manly BFJ (2006). Randomization, Bootstrap and Monte Carlo Methods in Biology. Statistical Science Series.

[B75] Stevens PF (2001). Angiosperm Phylogeny Website. Version 7, May 2006. http://www.mobot.org/MOBOT/Research/APweb/welcome.html.

[B76] Simon JL (1997). Resampling: The New Statistics.

[B77] Noyes HA, Morrison DA, Chance ML, T EJ (2000). Evidence for a neotropical origin of Leishmania. Memórias do Instituto Oswaldo Cruz.

[B78] Joy DA, Feng X, Mu J, Furuya T, Chotivanich K, Krettli AU, Ho M, Wang A, White NJ, Suh E, Beerli P, Su X (2003). Early origin and recent expansion of Plasmodium falciparum. Science.

[B79] Stermitz FR, Lorenz P, Tawara JN, Zenewicz L, Lewis K (2000). Synergy in a medicinal plant: antimicrobial action of berberine potentiated by 5'-methoxyhydnocarpin, a multidrug pump inhibitor. Proceedings of the National Academy of Sciences of the United States of America.

[B80] Raskin I, Ripoll C (2004). Can an apple a day keep the doctor away?. Current Pharmaceutical Design.

[B81] U.S. Department of Health and Human Services, Food and Drug Administration Guidance for Industry Botanical Drug Products. http://www.fda.gov/cder/guidance/1221dft.htm.

[B82] Balée W (2003). Historical-Ecological influences on the word for Cacao in Ka'apor. Anthropological Linguistics.

[B83] Lewis W, Elvin-Lewis M (2006). Distinguished Economic Botanist award lecture. Society of Economic Botany Annual Meeting.

[B84] Moerman DE, Estabrook GF (2003). Native Americans' choice of species for medicinal use is dependent on plant family: confirmation with meta-significance analysis. J Ethnopharmacol.

[B85] Avise JC, Johns GC (1999). Proposal for a standardized temporal scheme of biological classification for extant species. Proceedings of the National Academy of Sciences of the United States of America.

[B86] Richardson JE, Pennington RT, Pennington TD, Hollingsworth PM (2001). Rapid diversification of a species-rich genus of neotropical rain forest trees. Science (Washington D C).

[B87] Renner SS, Won H (2001). Repeated evolution of dioecy from monoecy in Siparunaceae (Laurales). Systematic Biology.

[B88] Sanderson MJ (1997). A nonparametric Approach to Estimating Divergence Times in the Absence of Rate Constancy. Molecular Biology and Evolution.

[B89] Clegg MT, Gaut BS, Learn GH, Morton BR (1994). Rates and patterns of chloroplast DNA evolution. Proceedings of the National Academy of Sciences of the USA.

[B90] Soltis DE, Soltis PS, Chase MW, Mort ME, Albach DC, Zanis M, Savolainen V, Hahn WH, Hoot SB, Fay MF, Axtell M, Swensen SM, Prince LM, Kress WJ, Nixon KC, Farris JS (2000). Angiosperm phylogeny inferred from 18S rDNA, rbcL, and atpB sequences. Botanical Journal of the Linnean Society.

[B91] Magallon S, Crane PR, Herendeen PS (1999). Phylogenetic pattern, diversity, and diversification of Eudicots. Annals of the Missouri Botanical Garden.

[B92] Soltis D Deep Time Project: A Comprehensive Phylogenetic Tree of Living and Fossil Angiosperms. http://www.flmnh.ufl.edu/deeptime/projectsummary.html.

[B93] Sun G, Ji Q, Dilcher DL, Zheng SL, Nixon K, Wang XF (2002). Archaeofruntaceae, a new basal angiosperm family. Science (Washington D C).

[B94] Purves WK, al. (1998). Life: The science of biology.

[B95] Fiehn O (2002). Metabolomics--the link between genotypes and phenotypes. Plant Molecular Biology.

[B96] Robinson T (1991). The Organic Constituents of Higher Plants.

[B97] Merken HM, Beecher GR (2000). Measurement of food flavonoids by high-performance liquid chromatography: A review. Journal of Agricultural and Food Chemistry.

[B98] Gostan T, Moreau C, Juteau A, Guichard E, Delsuc MA (2004). Measurement of aroma compound self-diffusion in food models by DOSY. Magnetic Resonance in Chemistry.

[B99] Delsuc MA, Malliavin TE (1998). Maximum Entropy Processing of DOSY NMR Spectra. Analytical Chemistry.

[B100] Harborne JL, Turner BL (1984). Plant Chemosystematics.

[B101] Judd W, Campbell C, Kellogg E, Stevens P (1999). Plant Systematics: A Phylogenic Approach.

[B102] Evans WC (1996). Trease and Evans' pharmacognosy.

[B103] Johns TA (1990). With bitter herbs they shall eat it : chemical ecology and the origins of human diet and medicine.

[B104] Shepard GH (1999). Pharmacognosy and the senses in two Amazonian. Anthroplogy.

[B105] Balunas MJ, Jones WP, Chin YW, Mi Q, Farnsworth NR, Soejarto DD, Cordell GA, Swanson SM, Pezzuto JM, Chai HB, Kinghorn AD (2006). Relationships Between Cytotoxicity, Plant Profiles, and Compound Classes Isolated in an Anticancer Drug Discovery Project. Chemistry and Biodiversity.

[B106] Isselbacher KJ (1980). Harrison's Principles of Internal Medicine.

[B107] Cookson WO (1999). Disease taxonomy--polygenic. British Medical Bulletin.

[B108] Specht CD (1996). Ethnocladistics: using cladistics to analyze ethnobotanical data. Joint meeting of the Society of Economic Botany and the International Society for Ethnopharmacology.

[B109] Specht CD (1997). Ethnocladistics: A predictive analysis of medicinal properties of plant families based on the ethnopharmacoepia of the Chacobo, Beni, Bolivia. Meetings of the International Society for Ethnobotany.

[B110] Lukhoba CW, Simmonds MSJ, Paton AJ (2006). Plectranthus: A review of ethnobotanical uses. Journal of Ethnopharmacology.

[B111] Senchina DS, Flagel LE, Wendel JF, Kohut ML (2006). Phenetic Comparison of Seven Echinacea Species Based on Immunomodulatory Characteristics. Economic Botany.

[B112] Daly DC, Stevenson DW (1998). Designing synergistic and intellectually equitable collaborations between biodiversity research and drug discovery investigations.. Second Monroe Wall Symposium.

[B113] Fujimaki Y, Kamachi T, Yanagi T, Caceres A, Maki J, Aoki Y (2005). Macrofilaricidal and microfilaricidal effects of Neurolaena lobata, a Guatemalan medicinal plant, on Brugia pahangi. J Helminthol.

[B114] Berger I, Passreiter CM, Caceres A, Kubelka W (2001). Antiprotozoal activity of Neurolaena lobata. Phytother Res.

[B115] Franssen FF, Smeijsters LJ, Berger I, Medinilla Aldana BE (1997). In vivo and in vitro antiplasmodial activities of some plants traditionally used in Guatemala against malaria. Antimicrob Agents Chemother.

[B116] Lentz DL, Clark AM, Hufford CD, Meurer-Grimes B, Passreiter CM, Cordero J, Ibrahimi O, Okunade AL (1998). Antimicrobial properties of Honduran medicinal plants. J Ethnopharmacol.

[B117] ProQuest LLC MEDLINE. http://medline.cos.com.

[B118] Lamb J, Crawford ED, Peck D, Modell JW, Blat IC, Wrobel MJ, Lerner J, Brunet JP, Subramanian A, Ross KN, Reich M, Hieronymus H, Wei G, Armstrong SA, Haggarty SJ, Clemons PA, Wei R, Carr SA, Lander ES, Golub TR (2006). The Connectivity Map: using gene-expression signatures to connect small molecules, genes, and disease. Science.

[B119] Cavalli-Sforza LL, Menozzi P, Piazza A (1994). The history and geography of human genes.

[B120] Foster M, Sturtevant WC (1996). Language and the culture history of North america. Handbook of North American Indians.

[B121] Nichols J, Durham WH (1997). Modeling ancient population structures and movement in linguitsics. Annual Review of Anthropology, vo 26.

[B122] Underhill PA, Shen P, Lin AA, Jin L, Passarino G, Yang WH, Kauffman E, Bonné-Tamir B, Bertranpetit J, Francalacci P, Ibrahim M, Jenkins T, Kidd JR, Mehdi SQ, Seielstad MT, Wells RS, Piazza A, Davis RW, Feldman MW, Cavalli-Sforza LL, Oefner PJ (2000). Y chromosome sequence variation and the history of human populations. Nature Genetics.

[B123] Wells S (2006). Deep ancestry : inside the Genographic Project.

[B124] Duke J Phytochemical and Ethnobotanical Databases. http://www.ars-grin.gov/duke/.

[B125] Moerman DE Native American Ethnobotany Database: Foods, Drugs, Dyes, and Fibers of Native North American Peoples. http://herb.umd.umich.edu/.

[B126] Skoczen S, Bussmann RW (2006). ebDB - Filling the gap for an International Ethnobotany Database. Lyonia.

